# An Exploration of Molecular Correlates Relevant to Radiation Combined Skin-Burn Trauma

**DOI:** 10.1371/journal.pone.0134827

**Published:** 2015-08-06

**Authors:** Aminul Islam, Svetlana Ghimbovschi, Min Zhai, Joshua M. Swift

**Affiliations:** 1 Radiation Combined Injury Program, Armed Forces Radiobiology Research Institute, Bethesda, Maryland, United States of America; 2 Children’s National Medical Center, Department of Integrative Systems Biology, Washington DC, United States of America; 3 Naval Medical Research Center, Undersea Medicine Department, Silver Spring, Maryland, United States of America; Oxford Brookes University, UNITED KINGDOM

## Abstract

**Background:**

Exposure to high dose radiation in combination with physical injuries such as burn or wound trauma can produce a more harmful set of medical complications requiring specialist interventions. Currently these interventions are unavailable as are the precise biomarkers needed to help both accurately assess and treat such conditions. In the present study, we tried to identify and explore the possible role of serum exosome microRNA (miRNA) signatures as potential biomarkers for radiation combined burn injury (RCBI).

**Methodology:**

Female B6D2F1/J mice were assigned to four experimental groups (n = 6): sham control (SHAM), burn injury (BURN), radiation injury (RI) and combined radiation skin burn injury (CI). We performed serum multiplex cytokine analysis and serum exosome miRNA expression profiling to determine novel miRNA signatures and important biological pathways associated with radiation combined skin-burn trauma.

**Principal Findings:**

Serum cytokines, IL-5 and MCP-1, were significantly induced only in CI mice (p<0.05). From 890 differentially expressed miRNAs identified, microarray analysis showed 47 distinct miRNA seed sequences significantly associated with CI mice compared to SHAM control mice (fold change ≥ 1.2, p<0.05). Furthermore, only two major miRNA seed sequences (miR-690 and miR-223) were validated to be differentially expressed for CI mice specifically (fold change ≥ 1.5, p<0.05).

**Conclusions:**

Serum exosome miRNA signature data of adult mice, following RCBI, provides new insights into the molecular and biochemical pathways associated with radiation combined skin-burn trauma *in vivo*.

## Introduction

Accidental or malicious exposure to high dose radiation combined with many kinds of other injuries, ranging from physical wounds, thermal burns to infection, often results in a negative synergistic response more harmful than the sum of the individual injuries [1,2]. The practical consequences of radiation combined injury, as well as an understanding that the body’s response to radiation combined trauma may be different from the responses to radiation or physical injury alone, needs to be better understood. Human studies have demonstrated that burns and wounds complicate the morbidity and mortality of personnel exposed to ionizing radiation and ultimately contribute to increased susceptibility to infection and higher mortality when compared to radiation-only injuries [1,3]. The mechanisms of these interactions are not fully understood and no evidence-based guidelines exist for rehabilitation or recovery of individuals with such type of injuries. As radiation combined burn injuries are expected to be prevalent in a possible radiation/nuclear scenario, knowledge on reliable biochemical and molecular biomarkers which can be used to accurately assess and implement effective countermeasures for such injury is critical for future emergency planning and national preparedness.

Current approaches to estimate radiation dose exposure and subsequent medical consequences rely on post-exposure techniques such as lymphocyte depletion kinetics, clinical observation, and the dicentric chromosome assay [4–6]. Such processes are cumbersome requiring highly technical and labor intensive procedures. What is needed is a novel biomarker which can be robust and stable, repeatedly assayable, and assessed using minimally invasive or non-invasive techniques. One such possibility could be the use of microRNA (miRNA) signatures derived from peripheral blood samples.

Small non-coding RNA known as miRNA have been shown to be important regulators of gene expression [7]. *In vivo* and *in vitro* studies have found that miRNA control is essential for the proper execution of many processes in normal cells, including cell metabolism, cell differentiation, and cell signaling [8,9]. Recent findings demonstrate that radiation has a significant impact on miRNA expression [10]. In addition, microRNA expression patterns have reflected the pathophysiological status of a tissue and have been shown to be specific for particular disease states [11,12]. Furthermore, miRNAs can be sourced from easily accessible body fluids such as blood derivatives and have been shown to be present as a stable and reliable reservoir within serum exosomes [13].

In this study, we investigate the potential of serum exosome miRNA signatures to be used as biomarkers for radiation combined skin-burn injury (RCBI) in a proof-of-principle *in vivo* study utilizing a mouse model of combined radiation skin burn injury (CI) and miRNA microarray analyses. In this work, we showed that RCBI induces specific serum cytokine pathways and has significant correlation to two distinct miRNA signatures. Our current study presents these miRNA signatures as possible biomarkers for RCBI and highlights targeted pathways that could possibly be manipulated to both better diagnose and treat patients subjected to radiation combined skin-burn trauma.

## Materials and Methods

### Animal studies

B6D2F1/J female mice (Jackson Laboratory, Bar Harbor, ME) at 12 to 16 weeks of age were maintained in an animal facility accredited by the Association for the Assessment and Accreditation of Laboratory Animal Care (AAALAC) in plastic micro-isolator cages with hardwood chip bedding. Commercial rodent chow and acidified potable water were provided ad libitum. Animal holding rooms were maintained at 20–26°C with 30–70% relative humidity using at least 10 changes per hour of 100% conditioned fresh air. A 12 h 06:00 (light) to 18:00 (dark) full-spectrum lighting cycle was in operation for the holding room. Mice were assigned into four injury groups: sham (n = 20), burn injury (n = 20), radiation injury (n = 20) and radiation combined burn injury (n = 22). The Armed Forces Radiobiology Research Institute (AFRRI) Institutional Animal Care and Use Committee (IACUC) reviewed and approved all animal procedures involved in this experiment.

Endpoints for the animal survival study were determined when there was death from the sequelae of CI or euthanasia for moribund mice or survivors after mitigation of the sequelae of RI and CI. To minimize animal pain or distress, moribund mice were considered to have arrived at the study endpoint. Such animals were euthanized by CO2 inhalation followed by cervical dislocation. Mice that survive for more than 30 days following irradiation were also euthanized using CO2 inhalation followed by cervical dislocation and used for subsequent cytokine and microarray analysis. Animals were scored twice daily throughout the survival study using an IACUC approved Rodent Intervention Score Sheet to assess their wellbeing and clinical status. To minimize animal pain the analgesics acetaminophen and buprenorphine were administered when appropriate and procedures were conducted under methoxyflurane anesthesia to reduce pain and distress. All study endpoints were approved by the IACUC.

### Gamma irradiation

Mice were given 9.5 Gy (LD70/30 for RCBI) whole-body bilateral ^60^Co gamma-photon radiation delivered at a dose rate of 0.4 Gy/min, while held in vertically stacked, ventilated, four-compartment, acrylic plastic boxes that provided electron equilibrium during irradiation. Empty compartments within the boxes were filled with 7.5 x 2.5 cm acrylic phantoms to ensure uniform electron scattering. The mapping of the radiation field was performed with alanine/EPR dosimetry [14]. The mapping provided dose rates to water within the core of the acrylic phantoms in each compartment of the mouse rack on that specific day. The field was uniform within ± 1.8% over all of the 120 compartments. Calibration of the dose rate with alanine was traceable to the National Institute of Standards and Technology (NIST) and the National Physics Laboratory of the United Kingdom. Sham-irradiated mice were placed in the same acrylic restrainers, taken to the radiation facility, and restrained for the time required for irradiation.

### Skin-burn injury

Skin surface burn injuries were performed on the shaved dorsal surface of mice. Animals receiving skin burns were anesthetized by methoxyflurane inhalation. A 15% total body-surface-area skin burn was performed within 1 h of irradiation using a 2.5 x 2.5 cm custom designed metal template positioned centrally over the shaved dorsal skin surface. Mice received a 12 second burn from ignited 95% ethanol (0.5 mL) [8]. All mice subjected to the skin burn injury and their controls were administered 0.5 mL sterile 0.9% saline intraperitoneally (i.p.) which contained analgesics, acetaminophen (150 mg/kg, Cadence Pharmaceuticals, CA) and buprenorphine (0.05 mg/kg), immediately after skin burn injury to alleviate pain. Four hours post-injury mice were given a second dose of acetaminophen (150 mg/kg, i.p.). Additional analgesic doses were considered during the 30 day study duration.

### Survival monitoring and blood collection

Animals were monitored at least twice daily for their general health and survival for 30 days. Mice body weights were measured on days 0, 1, 3, 7, 14, 21 and 28. Mice water consumption levels were assessed from days 1 to 7. On day 30, all surviving mice were anesthetized by isoflurane inhalation. Blood samples were collected by cardiac puncture followed by cervical dislocation. Serum was isolated from whole blood for analysis using the Capiject blood collection system tubes (Terumo, Elkton, MD).

### Exosome Western blot analysis

Exosome lysates from the SeraMir Exosome RNA assay were subjected to denaturing and reducing gel electrophoresis, and electrophoretic blotting as described [15]. Alix, Resistin, and TSG101 serum exosome protein markers [16,17] were detected using mouse primary anti–Alix,–Resistin, and–TSG101 antibodies (Santa Cruz Biotechnology Inc., Dallas, TX) diluted 1:200 respectively and an appropriate horseradish peroxidase-conjugated IgG secondary antibody (GE Life Science, Piscataway, NJ) diluted 1:1000.

### Microarray and data analysis

MicroRNA from serum exosome was isolated and amplified from 250 μl mice serum samples using SeraMir Exosome RNA Amplification kit (System Biosciences, Mountain View, CA) according to manufacturer’s instructions. RNA quality was assessed using Agilent 2100 Bioanalyzer (Agilent Technologies Inc., Santa Clara, CA).

For miRNA expression profiling we used Affymetrix Gene-Chip microRNA Arrays approach, as described by the manufacturer (Affymetrix, Santa Clara, CA) and previously [15]. An aliquot of 300 ng of the enriched and quantitated miRNA was biotin-labeled with FlashTag Biotin HSR RNA Labeling Kit. Following biotin labeling, quality confirmation was performed using the Enzyme Linked Oligosorbent Assay (ELOSA) protocol (Affymetrix). 21.5 μl of each high-quality biotin-labeled miRNA sample was hybridized to Affymetrix Gene-Chip miRNA 3.0 Array for 16 h according to manufacturer’s protocol. The arrays were washed and stained on the Affymetrix Fluidics station 400 and scanned with a Hewlett Packard G2500A gene Array Scanner.

Affymetrix Expression Console software was used for the microarrays data generation, normalization and quality control (QC). To generate miRNA expression values Affymetrix GeneChip derived CEL intensity files were analyzed using Affymetrix Expression Console normalization probe set algorithm for miRNA: RMA+DABG (Robust Multi-array Average plus Detection Above the Background). To evaluate the success of the labeling protocol and array processing, five spike-in control probe sets signal values were checked for expression levels (signals ≥ 1,000 were considered as passed for QC).

Expression Console results table was used in Hierarchical Clustering Explorer (HCE) v.3 for probe set filtering, power analysis and Chip-based unsupervised clustering [18]. HCE unsupervised cluster analysis revealed two control samples as outliers, which were removed out of the study, leaving a total of 22 samples for the analysis. Expression Console derived CHP files, containing miRNAs signal intensity values after RMA+DABG, were used in Partek Genomics Suite, version 6.5 (Partek, St. Louis, MO) to determine differently expressed miRNAs, statistics and data visualization. Using Partek, gene expression signal values were filtered based on average signal values and 20% of the probe sets (miRNAs) with the lowest signal values were removed and not included for further analysis. One-Way ANOVA statistical test was applied to verify significance of the comparative results. Only miRNAs having expression signal values with a p value <0.05 and fold change ≥ 1.5 were considered for further analyses. All original microarray data are deposited in the NCBI GEO database (accession number: GSE66739).

### Pathway and network analysis

To determine meaningful molecular networks and pathways we used the Ingenuity Pathways Analysis (IPA) software application (QIAGEN, Valencia, CA). IPA generates networks, where differentially regulated genes can be related according to previously known associations between miRNA and their mRNA targets. All generated networks are ordered by a score meaning significance. MicroRNA–mRNA integration gene lists and networks have been created and analyzed after using the IPA Integration Tool, which determined mRNA targets (experimentally observed and highly predicted) for statistical significant miRNA seed sequences derived from Partek. The Core Analysis and Pathway Build (Grow) IPA functions were used to interpret miRNA and their mRNA targets in the context of biological processes, pathways and networks.

### Cytokine multiplex assays

Serum cytokine concentrations were evaluated using the Bio-Plex Pro Mouse Cytokine Group I 23-plex Assay kit (Bio-Rad, Hercules, CA) following the manufacturer’s directions. Samples were analyzed using the Luminex Bio-Plex 200 System and data software package (Bio-Rad, Hercules, CA).

### Quantitative RT-PCR

Relevant TaqMan microRNA Assay probes were obtained from Life Technologies and used per manufacturers’ instructions (Grand Island, NY). Real time RT-PCR was performed for 40 cycles using the Bio-Rad iCycler iQ real time PCR thermocycler and iScript SYBR green PCR supermix (Hercules, CA). Quantification of the RT-PCR products normalized to glyceraldehyde-3-phosphate dehydrogenase (GAPDH) expression was performed using iCycler iQ data analysis software and comparative CT method.

### Data processing and statistical analysis

Non-microarray data are expressed as mean ± SEM. Data sets were analyzed by one way ANOVA with a Bonferroni correction for multiple comparisons. The results were considered significant at p ≤ 0.05.

## Results

### Survival and serum cytokine profile of RCBI in mice

**Fig 1:**
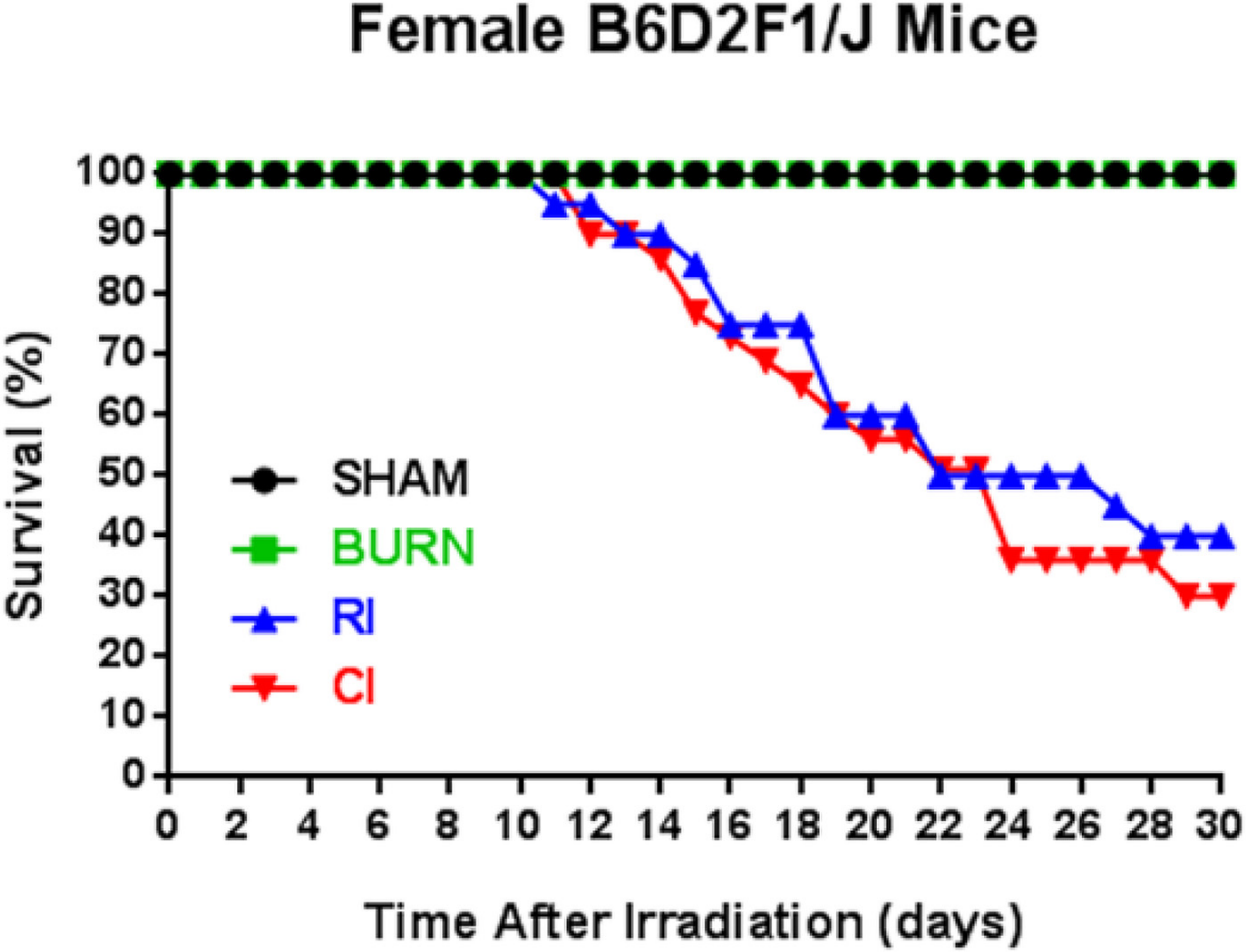
Survival profile associated with sham, burn, RI and CI mice over 30 days. 30-day survival analysis for female B6D2F1/J mice subjected to skin burn injury alone (BURN), whole-body ionizing irradiation alone (RI) or when combined with skin burn injury (CI) compared to experimental control mice (SHAM), n = 20–22 per group.

**Fig 2:**
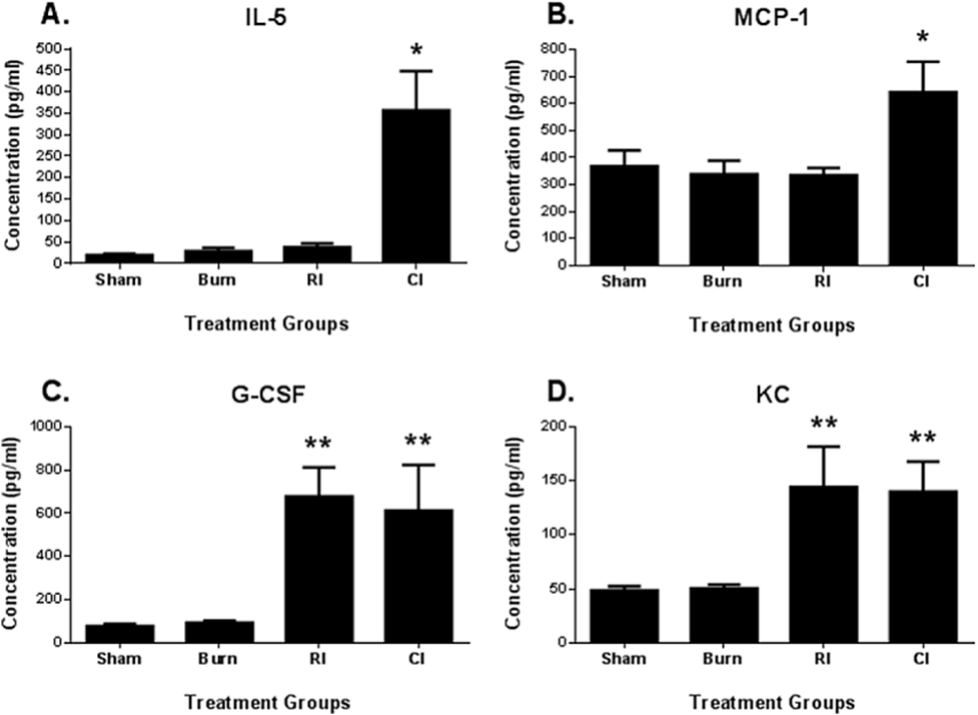
IL-5, MCP-1, G-CSF and KC cytokine levels in serum of sham, burn, RI and CI mice. Analysis performed using cytokine multiplex assays to assess **(A)** IL-5, **(B)** MCP-1, **(C)** G-CSF and **(D)** KC serum levels in female B6D2F1/J mice subjected to skin burn injury alone (BURN), whole-body ionizing irradiation alone (RI) or when combined with skin burn injury (CI) compared to experimental control mice (SHAM) at 30 days survival. *p<0.05 for differences between sham, burn and RI mice groups (mean ± SEM, n = 6). **p<0.05 for differences between sham and burn mice groups (mean ± SEM, n = 6).

Animal survival was evaluated over a 30-day period (Fig 1). Experimental control mice (SHAM) and mice subjected to skin burn injury alone (BURN) did not result in mortality over the 30-day observation period. Mice subjected to radiation injury alone (RI) decreased survival to 40% which was slightly higher than mice subjected to combined radiation skin burn injury (CI) which had a mean survival of 30% at day 30. As determined by cytokine multiplex assays, CI mice only had significantly higher IL-5 (Fig 2A) and MCP-1 (Fig 2B) serum cytokine levels compared to other groups (IL-5: 832%, p<0.05; MCP-1: 75%, p<0.05). Both RI and CI mice respectively had significantly higher G-CSF (Fig 2C) and KC (Fig 2D) serum cytokine levels compared to the remaining groups (G-CSF: 631 and 562%, p<0.05; KC: 185 and 176%, p<0.05).

### MicroRNA microarray expression analysis of RCBI in mice

**Fig 3:**
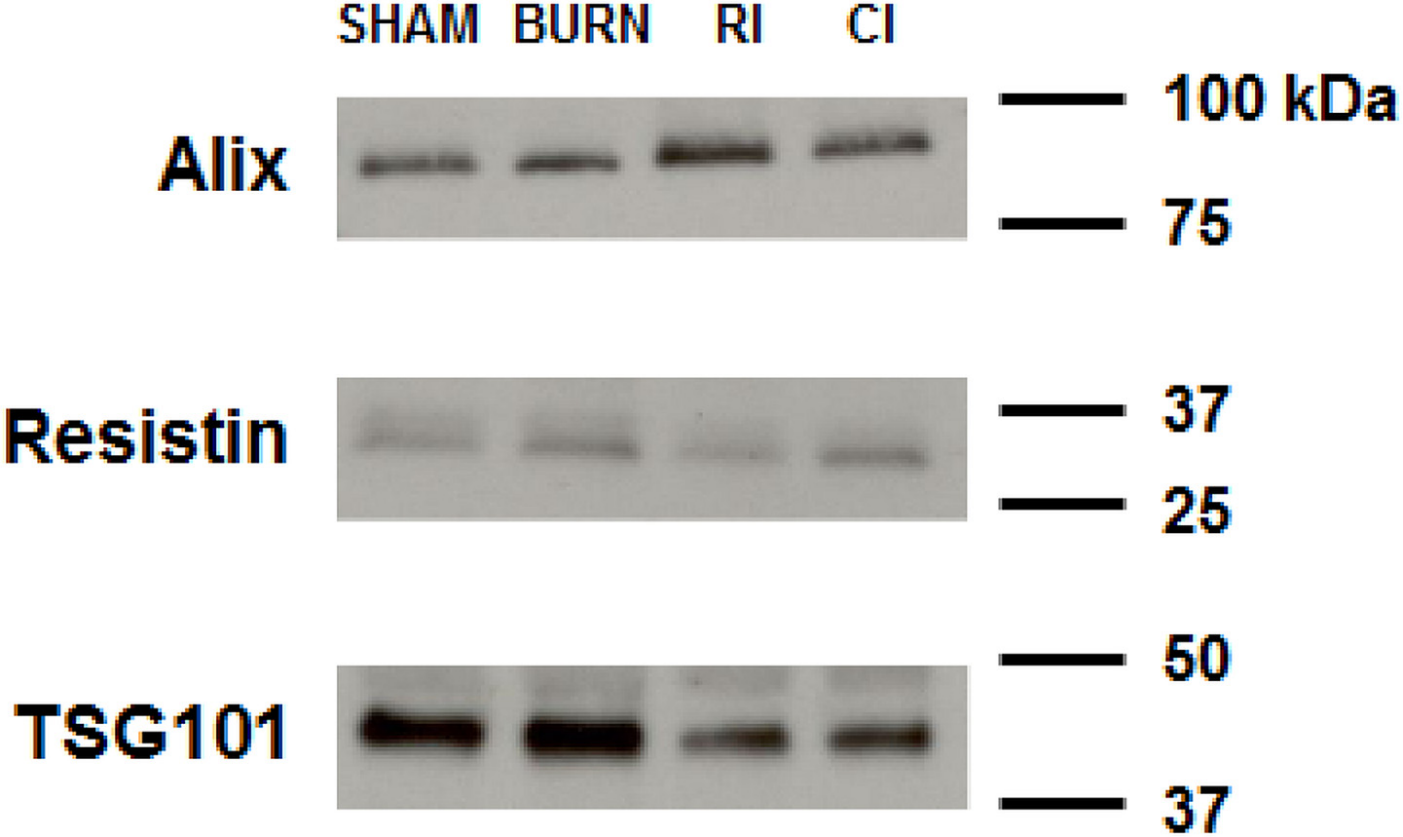
Western blot characterization of isolated serum exosomes used for extracting microRNA. Representative blots showing the expression of serum exosome protein markers: Alix, Resistin, and TSG101 in isolated exosome samples of mice subjected to skin burn injury alone (BURN), whole-body ionizing irradiation alone (RI) or when combined with skin burn injury (CI) compared to experimental control mice (SHAM) at 30 days survival.

**Fig 4:**
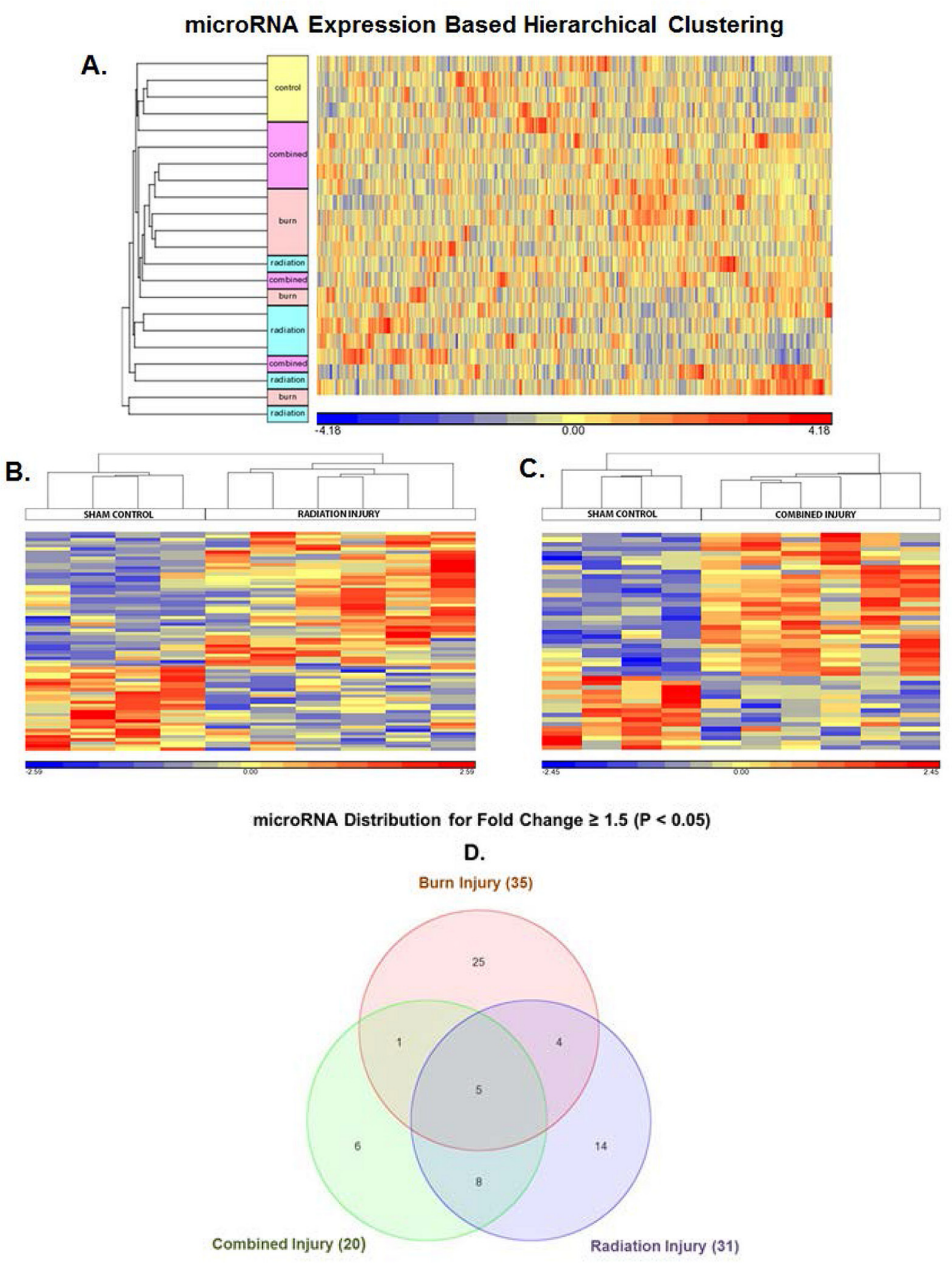
Identification of differentially expressed microRNA in serum exosomes of burn, RI and CI mice vs. control sham mice. **(A)** Heat Map analysis of microarray data from female B6D2F1/J mice subjected to skin burn injury alone (BURN), whole-body ionizing irradiation alone (RI) or when combined with skin burn injury (CI) compared to experimental control mice (SHAM) at 30 days survival. Here all miRNAs that significantly changed with a fold change ≥ 1.2 were included in the analysis (p<0.05, n = 6). Results were generated using Partek Genomics Suite. The color code for the signal strength in the classification scheme is shown in the panel below. Induced genes are indicated by shades of red and repressed genes are indicated by shades of blue. **(B)** Heat Map showing variations in expression of 70 miRNAs in RI mice vs. control sham mice using a cut-off criteria of p<0.05 and fold change ≥ 1.2. **(C)** Heat Map showing variations in expression of 47 miRNAs in CI mice vs. control sham mice using a cut-off criteria of p<0.05 and fold change ≥ 1.2. **(D)** Partek Venn-Diagram showing differential miRNA distribution within the different mice injury groups vs. control sham mice using a cut-off criteria of p<0.05 and fold change ≥ 1.5.

Following characterization of the isolated serum exosome samples using Western blot analysis and confirmation with known protein exosome markers [16,17] (Fig 3), a microarray analysis comparing serum exosome miRNA from BURN, RI and CI mice to experimental control SHAM mice (n = 6 per group) was conducted. 890 distinct miRNA seed sequences were identified as being significantly (p<0.05) different in all groups compared to SHAM mice (Fig 4A and S1 Table). All differentially expressed miRNA sequences significantly (p<0.05) specific to the individual injury groups: BURN, RI and CI compared to SHAM are presented in S2–S4 Tables. The majority of the miRNAs (80%) had less than a 1.5-fold difference and only about 4% of miRNAs had more than a two-fold difference in expression levels. 70 miRNA sequences from the RI mice (Fig 4B), 47 miRNA sequences from the CI mice (Fig 4C) and 90 miRNA sequences from the BURN mice were selected based on fold change and significance criteria (p<0.05 and fold change ≥ 1.2). From these groups a Partek Venn-Diagram model, using a cut-off criteria of p<0.05 and fold change ≥ 1.5, was applied to select miRNA sequences specific to BURN, RI and CI mice (Fig 4D). This revealed 14 and six miRNA sequences specific only to RI and CI mice respectively. From this list we identified two major miRNA sequences for CI mice (Table 1) and nine major miRNA sequences for RI mice (Table 2). These 11 miRNA seed sequences were validated using real-time quantitative RT-PCR.

**Table 1:** List of major microRNA seed sequences identified using microarray to be differentially expressed only in CI mice compared to SHAM mice with fold change ≥ 1.5 and p value of significance (p<0.05).

miRNA seed sequences	Microarray fold change	P value
miR-690	1.8	0.0485
miR-223	1.6	0.0084

**Table 2:** List of major microRNA seed sequences identified using microarray to be differentially expressed only in RI mice compared to SHAM mice with fold change ≥ 1.5 and p value of significance (p<0.05).

miRNA seed sequences	Microarray fold change	P value
miR-34b-3p	2.0	0.0429
miR-3082-5p	2.0	0.0344
miR-142-5p	-1.5	0.0096
miR-31	-1.5	0.0026
miR-185	-1.6	0.0094
miR-130b	-1.7	0.0296
miR-216b	-1.7	0.0054
miR-130a	-1.9	0.0171
miR-1912	-2.1	0.0204

### MicroRNA expression validation using quantitative RT-PCR

**Fig 5:**
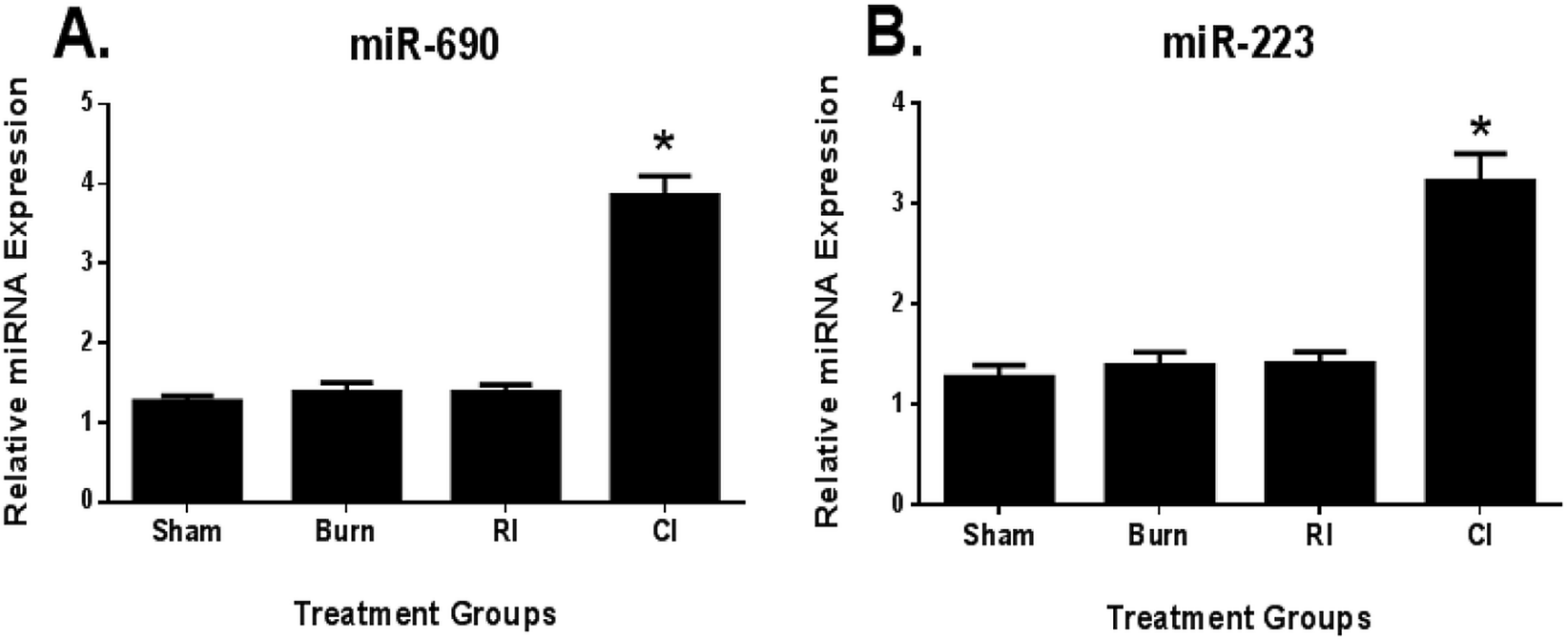
Validation of differentially expressed microRNA in serum exosomes relevant to CI pathways in mice using quantitative RT-PCR. Expression of target miRNA seed sequences **(A)** miR-690 and **(B)** miR-223 determined to be important in radiation combined skin burn injury pathways of female B6D2F1/J mice subjected to skin burn injury alone (BURN), whole-body ionizing irradiation alone (RI) or when combined with skin burn injury (CI) compared to experimental control mice (SHAM) at 30 days survival. *p<0.05 for differences between sham, burn and RI mice groups (mean ± SEM, n = 6).

**Fig 6:**
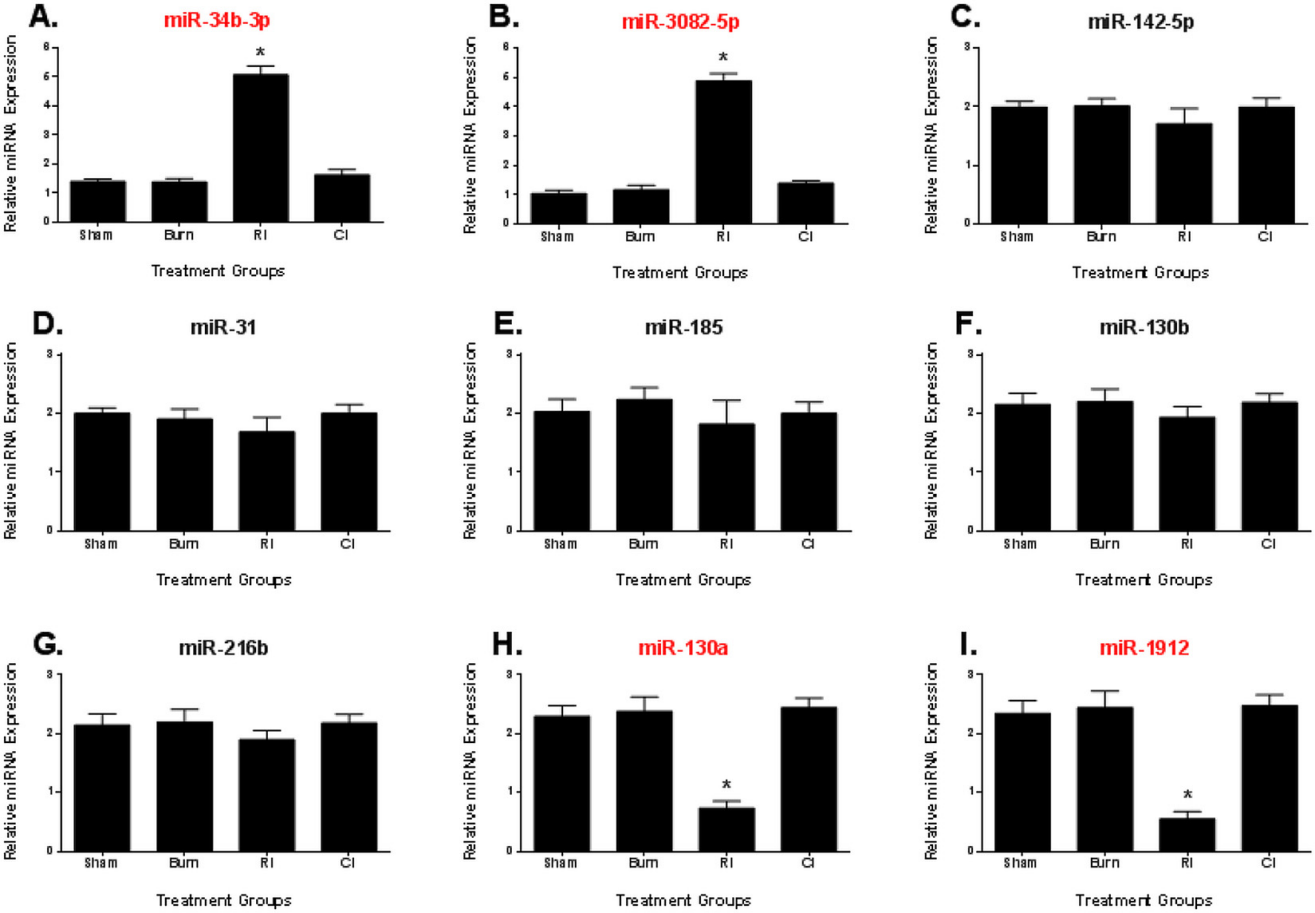
Validation of differentially expressed microRNA in serum exosomes relevant to RI pathways in mice using quantitative RT-PCR. Expression of target miRNA seed sequences **(A)** to **(I)** determined to be important in radiation injury pathways of female B6D2F1/J mice subjected to skin burn injury alone (BURN), whole-body ionizing irradiation alone (RI) or when combined with skin burn injury (CI) compared to experimental control mice (SHAM) at 30 days survival. *p<0.05 for differences between sham, burn and CI mice groups (mean ± SEM, n = 6).

From the microarray analysis derived sequences, both the miRNA seed sequences specific to CI mice were successfully validated for differential expression using quantitative RT-PCR as shown in Fig 5 (miR-690 and miR-223). In contrast, only four miRNA seed sequences specific to RI mice were successfully validated for differential expression using quantitative RT-PCR as shown in Fig 6 (miR-34b-3p, miR-3082-5p, miR-130a and miR-1912). This highlighted the essential need of validating all expression data findings obtained from microarray experiments.

### Analysis of molecular pathways important for RCBI in mice

**Fig 7:**
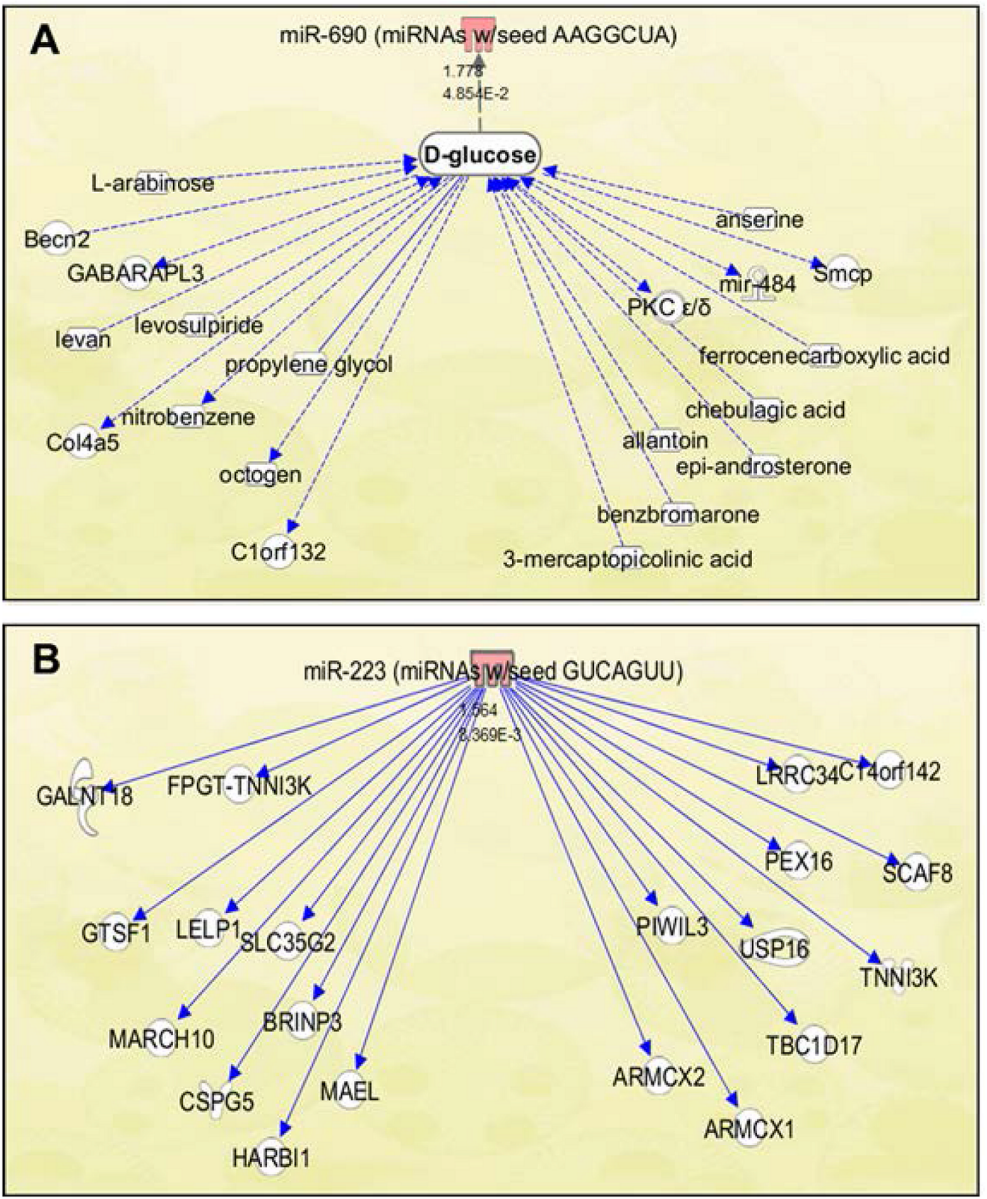
Molecular gene networks in mice associated with serum exosome microRNA sequences relevant to CI pathways. Illustrated IPA (Ingenuity Pathways Analysis) generated gene pathways associated with miRNA seed sequences **(A)** miR-690 and **(B)** miR-223. Relationships are primarily due to co-expression, but may include phosphorylation/dephosphorylation, proteolysis, transcription, binding, inhibition, activation/deactivation, and biochemical modification. Indirect connections are shown for miR-690 via D-glucose as no direct gene pathways were found using IPA. Top 20 connections for both miRNA sequences were provided as determined using IPA.

Using IPA software, gene pathway analysis was performed on the two miRNA seed sequences most relevant to CI mice in our study. The IPA-generated gene networks for the top 20 connections associated with miRNA seed sequences miR-690 and miR-223 is illustrated in Fig 7. Interestingly, directly associated disease or biological function pathways could not be identified for miR-690 suggesting it to be unique for RCBI. Instead indirect gene pathways were assembled via D-glucose for miR-690 (Fig 7A). In contrast, directly associated disease and biological function pathways were identified for miR-223 and involved gene pathways mainly associated with kidney disease, cancer, immunological disease, and biological processes associated with cell death and survival (Fig 7B).

## Discussion

Appropriate medical interventions needed to treat individuals with RCBI are unavailable and biological mechanisms related to these types of injury are poorly understood. In our study, we analyzed microRNA expression in peripheral blood serum exosomes of mice subjected to radiation combined skin-burn trauma using whole body irradiation at a dose of 9.5 Gy. Our main finding is the identification of distinctive serum miRNA signatures which are specific to RCBI only, using our combined radiation-injury model. A practical outcome of our study is that it could possibly lead to the future use of miRNA expression signatures as biomarkers in the development of diagnostic and prognostic tools for individuals subjected to RCBI.

Single stranded, non-protein coding, small RNAs known as miRNAs have emerged as critical regulators of cell differentiation, identity and maintenance [19]. As part of the RNA-induced silencing complex (RISC) miRNAs target mRNA transcripts mainly within the 3’UTR to promote mRNA degradation and/or translational repression [20]. Nucleotides 2–8 from the 5’ end of the mature miRNA (seed sequence region) are important for targeting mRNA [21]. Each miRNA can target up to hundreds or thousands of mRNAs *in vivo* and therefore potentially regulate multiple biological pathways [22]. It has been suggested that miRNA, within body fluids, act as biomarkers for various physiological responses and pathological stages [23–25]. MicroRNAs are relatively stable in body fluids such as serum due to their smaller size and by being protected within exosomes [26]. It is thought that miRNA isolation from exosome improves the sensitivity of miRNA amplification from body fluids, and exosomal miRNA should form the basis for early biomarker studies using body fluids so as to incorporate all low abundance circulating miRNAs which are concentrated in exosomes [13]. This was the approach that we adopted in our study.

The role of exosomes in radiation response has been studied extensively at the *in vitro* level where radiation exposed cells produced exosomes which mediated genomic damage of un-irradiated (bystander) cells implicating a role for RNA [27,28]. Studies using the bystander effect have further implicated the contents of exosomes, particularly the RNA and protein cargo, to work in a synergistic manner to produce non-targeted results of genomic instability and bystander effect [29]. There have also been previous studies which have used similar approaches to our group in characterizing miRNA signatures in radiation response to identify novel pathways involved in cellular stress response to radiation exposure with the aim of unearthing new insight into the biological roles of miRNAs [30,31].

An urgent need to develop non-invasive biomarkers reflective of radiation-exposure related injuries based on molecular changes exists. Previous attempts at molecular biodosimetry involved using mRNA expression, from isolated mononuclear cells, to predict the effects of radiation exposure; however, mRNA is known to be intrinsically unstable thus making this method unreliable [32]. Plasma DNA level measurements have been also judged to be unreliable for this purpose due to its non-specific fluctuations during the passage of several different illnesses [33]. The most promising is the blood protein-based approach, which was supported in our study by the identification of serum protein cytokines IL-5 and MCP-1 to be specifically associated with RCBI animals and requires further investigation in terms of their biological significance. Both play important roles in hematopoietic functions as IL-5 is known to increase immunoglobulin secretion and is a key mediator in eosinophil activation [34], while MCP-1 is involved in regulating the migration and infiltration of monocytes/macrophages [35]. Such protein based approaches have many challenges in terms of the complexity of protein compositions, low abundance of proteins of interest, post-translational modifications, and difficulty in developing detection methods with high affinity [36]. As a result we additionally pursued the miRNA microarray approach in identifying novel biomarkers for RCBI.

The feasibility of using blood miRNA as *in vivo* biomarkers is supported by their predicted ability to regulate the expression of more than 50% of the human protein coding genes by means of mRNA destabilization and translational repression [9] and playing major roles in cell signaling pathways, physiological processes, and human pathologies [37–39]. Furthermore, miRNA are relatively stable and easily accessible in many biological fluids [13]. Plasma miR-122 has been shown to be increased by various forms of hepatic diseases [40]. Both miR-208b and miR-449 have been shown to be highly elevated by cardiovascular damage [41], while several plasma miRNAs have been shown to be specifically affected by drug-induced liver damage [42]. Our miRNA expression microarray and validation experiments, conducted on serum exosome samples, identified four miRNA seed sequences that corresponded significantly to RI mice (miR-34b-3p, miR-3082-5p, miR-130a and miR-1912), and two miRNA seed sequences that corresponded significantly to CI mice (miR-690 and miR-223). Previous *in vivo* studies have used radiation-only exposure protocols to assess miRNA utility as a peripheral blood derived biomarker [43–45], and as a result makes our combined approach of examining miRNA expression, in both CI and RI animal models, unique and more realistic in the sense that radiation-combined injuries are expected to be prevalent in future radiation/nuclear scenarios as demonstrated by the recent Fukushima Daiichi incident. As a result, such information is critical to future emergency planning and national preparedness with respect to medical triage and countermeasure development. Previously described blood miRNA signatures such as miR-21 [44], miR-142 [43] and miR-150 [45] have been proposed as potential biomarkers for exposure to ionizing radiation. These miRNA seed sequences do not match the ones which were experimentally determined by our study for RI. This could be explained by the fact that miRNA signatures induced by ionizing radiation in peripheral blood involves a highly complex process which is both radiation type- and radiation dose-specific [46,47]. Therefore, the potential utility of our miRNA signatures for RI requires further characterization.

The distinct miRNA signatures determined by our study for CI are considered to be novel and suggested to be specific for radiation combined skin-burn trauma only. Signature miR-690 was determined by Ingenuity Pathway Analysis to have no direct associations with other gene pathways. However, previous studies have linked miR-690 dysregulation to hematopoietic differentiation processes in mouse granulocytes [48], cannabinoid-induced immunosuppression regulation in myeloid cells [49], and susceptibility to testosterone and high glucose treatment in liver and pancreatic cells respectively [50,51]. In comparison, signature miR-223 was shown to have direct associations with several gene pathways using IPA and its dysregulation has been recently associated with human biological processes including: disease activity for psoriasis in peripheral blood mononuclear cells [52], biomarker for colorectal cancer screening using fecal blood samples [53], pathogenesis of acute ischemic stroke using peripheral blood samples [54], and biomarker of autosomal dominant polycystic kidney disease progression using urine samples [55]. To fully evaluate how both of these miRNAs specifically relate to as potential molecular biomarkers in serum exosome for RCBI, their individual roles within the pathways relevant to radiation combined skin-burn trauma must be studied. As mechanisms for RCBI and its interactions are not fully understood, a good starting point would be to closely investigate the pathways identified for these miRNAs using IPA as shown in Fig 7. Furthermore, *in vitro* studies which assess the functional consequences of our CI miRNA signatures in relation to regulating cytokine genes such as IL-5 and MCP-1 would prove to be valuable for further validation of our findings. Such studies are currently in progress in cooperation with our research collaborators.

In conclusion, our study provides a detailed investigation into the differentially expressed miRNAs and gene networks likely to be responsible for radiation combined skin-burn trauma *in vivo*. MicroRNA signatures such as miR-690 and miR-223 have been determined to act as potential molecular biomarkers for RCBI. Further studies are needed to confirm the importance of such miRNAs in the regulation of radiation-combined injuries. Ultimately, such information will lead to the targeted design of more effective strategies for both diagnosing and treating individuals, accidently or maliciously, subjected to radiation combined skin-burn trauma.

## Supporting Information

S1 NC3Rs ARRIVE List The ARRIVE guidelines checklist. (PDF)

S1 Table List of 890 microRNA seed sequences identified using microarray to be differentially expressed in BURN, RI and CI mice compared to SHAM control mice with respective fold change and p value of significance. (DOC)

S2 Table List of 106 microRNA seed sequences identified using microarray to be differentially expressed in BURN mice compared to SHAM mice with respective fold change and p value of significance (p<0.05). (DOC)

S3 Table List of 85 microRNA seed sequences identified using microarray to be differentially expressed in RI mice compared to SHAM mice with respective fold change and p value of significance (p<0.05). (DOC)

S4 Table List of 56 microRNA seed sequences identified using microarray to be differentially expressed in CI mice compared to SHAM mice with respective fold change and p value of significance (p<0.05). (DOC)
