# Supplementary material for: An Exploration of Molecular Correlates Relevant to Radiation Combined Skin-Burn Trauma
Source: PLoS One. 2015 Aug 6;10(8):e0134827. doi: 10.1371/journal.pone.0134827 (PMC4527694; doi:10.1371/journal.pone.0134827)
Supplement: S1 Table — (DOC) [file pone.0134827.s002.doc]

**S1 Table. List of 890 microRNA seed sequences identified using microarray to be differentially expressed in BURN, RI and CI mice compared to SHAM control** mice with respective fold change and p value of significance.

| **miRNA seed sequence**  **ID** | **Microarray**  **fold change**  **BURN vs. CON** | **P value** | **Microarray**  **fold change**  **RI vs. CON** | **P value** | **Microarray**  **fold change**  **CI vs. CON** | **P value** |
| --- | --- | --- | --- | --- | --- | --- |
| miR-688  let-7a-1-star  miR-871-3p  miR-331-3p  miR-126-5p  miR-130a  miR-452-3p  miR-767  miR-153  miR-376b-star  miR-30c-2-star  miR-466n-3p  miR-23b-star  miR-410  miR-1946a  miR-669e-star  miR-199b-star  miR-881  miR-298  miR-742-star  miR-196a-1-star  miR-467h  miR-713  miR-216b  miR-1298-star  miR-3096b-3p  miR-503  miR-574-5p  miR-3090  miR-1306-3p  miR-192-star  miR-31  miR-466h-3p  miR-383-star  miR-377  miR-495  miR-214  miR-350  miR-130b  miR-185  miR-148a  miR-466i-5p  miR-1186  miR-138-1-star  miR-1893  miR-125b-5p  miR-3065  miR-1264-5p  miR-199b  miR-429  miR-217-star  miR-3075  miR-376c-star  miR-302d-star  miR-183  miR-3072-star  miR-323-3p  miR-32-star  miR-26a-2-star  miR-196a-2-star  miR-293-star  miR-28c  miR-223  miR-680  miR-204-star  miR-17-star  miR-346  miR-1897-3p  miR-1196  miR-669l  miR-711  miR-691  miR-19a  miR-344b  miR-103-2-star  miR-34c  miR-467e-star  miR-3104-3p  miR-144-star  miR-130a-star  miR-3067-star  miR-181a  miR-666-3p  miR-669p-star  miR-34b-5p  miR-466q  miR-712-star  miR-1967  miR-491  miR-384-5p  miR-382-star  miR-763  miR-219-3p  miR-1905  miR-1962  miR-106a  miR-1839-3p  miR-344d-1-star  miR-3105-3p  miR-26b-star  miR-669b  miR-212-3p  miR-3094-star  miR-664-star  miR-20a-star  miR-5129  miR-5128  miR-107  miR-487b  miR-667  miR-497-star  miR-5116  miR-125b-2-3p  miR-465c-5p  miR-142-5p  miR-219-5p  miR-693-3p  miR-207  miR-302b  miR-133b  miR-3083  miR-532-5p  miR-381-star  miR-221-star  miR-1191  miR-1192  miR-135a  miR-1912-star  miR-1195  miR-574-3p  miR-668-star  miR-331-5p  miR-1897-5p  miR-1943  miR-154  miR-133a  let-7c-2-star  miR-3070b-5p  miR-466i-3p  miR-101b  miR-1955-5p  miR-683  miR-3068-star  miR-3969  miR-208b  miR-92a  miR-7a-1-star  miR-141  miR-450a-1-star  miR-3572  miR-1895  miR-1946b  miR-212-5p  miR-714  miR-3082-5p  miR-21-star  miR-211  miR-504-star  miR-3076-5p  miR-3475  miR-28b  miR-192  miR-344e-star  miR-5107  miR-3064-5p  miR-3100-3p  miR-499-star  miR-877-star  miR-494  miR-543  miR-487b-star  miR-3071-star  miR-669f-5p  miR-344  miR-700  miR-540-3p  miR-466l-3p  miR-455  miR-883a-5p  miR-669h-3p  miR-1904  miR-362-5p  miR-124  miR-1b-3p  miR-15a-star  miR-467b-star  miR-5117  miR-703  miR-451  miR-3961  miR-1912  miR-2183  miR-344e  miR-669b-star  miR-421-star  miR-25-star  miR-3087-star  miR-669j  miR-1941-3p  miR-503-star  miR-3083-star  miR-380-5p  miR-1957  miR-1188-star  miR-295-star  miR-669c  miR-875-3p  miR-744  miR-186  miR-3092  miR-195  miR-103-1-star  miR-122-star  miR-1982.1  miR-702  miR-669k  miR-1902  miR-103  miR-760-5p  miR-710  miR-1843-5p  miR-1950  miR-484  miR-3102  miR-32  miR-878-3p  miR-1892  miR-1249-star  miR-5112  miR-449b  miR-208a-5p  miR-3086-3p  miR-205  miR-26b  miR-465a-5p  miR-3073-3p  let-7a  miR-181a-2-star  miR-297a-5-star  miR-322  miR-3104-5p  miR-669o-5p  miR-3099-star  miR-3085-3p  miR-199a-3p  miR-5100  miR-708-star  let-7b-star  miR-350-star  miR-19b  miR-101a  miR-1249  miR-344d-2-star  miR-3963  miR-483-star  miR-466k  miR-3093-3p  miR-709  miR-20a  miR-5103  miR-1896  miR-701  miR-669m-3p  miR-3078  miR-496  miR-143-star  miR-466m-3p  miR-653-star  miR-466f-5p  miR-140-star  miR-466g  miR-211-star  miR-193-star  miR-376c  miR-505-3p  miR-99a-star  miR-101c  miR-742  miR-471-3p  miR-340-5p  miR-155-star  miR-344f-3p  miR-148a-star  miR-7b-star  miR-3102-star  miR-3061-3p  miR-690  miR-872  miR-344d  miR-34c-star  miR-489  miR-375-star  miR-669f-3p  miR-3096-3p  miR-365  miR-187-star  miR-3109-star  miR-1198-5p  miR-590-5p  miR-34b-3p  miR-467c-star  miR-18b-star  miR-883a-3p  miR-5136  miR-3098-3p  miR-29b  miR-467b  miR-200c-star  miR-9-star  miR-297a-star  miR-466d-3p  miR-3082-3p  miR-292-3p  miR-568  miR-664  miR-500-star  miR-1964-5p  miR-434-3p  miR-3964  miR-758-star  miR-3473d  miR-3102-3p.2  miR-106b-star  miR-208a-3p  miR-301a-star  miR-3072  miR-669c-star  miR-431-star  miR-3058  miR-3088  miR-16-2-star  miR-367-star  miR-202-3p  miR-93-star  miR-216b-star  miR-3471  miR-696  miR-34a-star  miR-720  miR-369-5p  miR-10a-star  miR-678  miR-190-star  miR-483  miR-293  miR-190b-star  miR-1969  miR-28-star  miR-705  miR-3091-3p  miR-328-star  miR-5127  miR-669o-3p  miR-695  miR-5114  miR-743b-5p  miR-463-star  miR-3470a  miR-298-star  miR-3105-5p  miR-196a  miR-344d-3-star  miR-467e  miR-541-star  miR-3077  miR-320  miR-17  miR-3968  miR-1894-5p  miR-98-star  miR-5099  miR-147-star  miR-190  miR-107-star  miR-30b  miR-3084-star  miR-743b-3p  miR-670  miR-452-5p  miR-5105  miR-5097  miR-143  miR-3058-star  miR-327  miR-3473  miR-654-3p  miR-485-star  miR-5104  miR-376b  miR-297a  miR-448-3p  miR-140  miR-23b  miR-21  miR-33-star  miR-7a  miR-374  miR-882  miR-185-star  miR-3108  miR-384-3p  miR-3069-3p  miR-341  miR-686  miR-3473c  miR-201-star  miR-365-1-star  miR-540-5p  miR-455-star  miR-3081-star  miR-1224  miR-200c  miR-141-star  miR-330-star  miR-1968  miR-126-3p  miR-1964-3p  miR-466f-3p  miR-31-star  miR-362-3p  miR-3472  miR-1907  miR-1306-5p  miR-5115  miR-1971  miR-669i  miR-501-3p  miR-470-star  miR-33  miR-290-3p  miR-301b-star  miR-187  miR-509-5p  miR-5106  miR-3066-star  miR-692  miR-340-3p  miR-208b-star  miR-329  miR-30c  miR-146b  miR-3097-3p  miR-153-star  miR-743a-star  miR-301a  miR-669l-star  miR-421  miR-701-star  miR-539-3p  miR-468  miR-98  miR-708  let-7i  miR-1899  miR-383  miR-322-star  miR-511-5p  miR-302d  miR-3473b  miR-193b  miR-2137  miR-1982.2  miR-429-star  miR-302c  miR-1949  miR-3064-3p  miR-592  miR-291b-5p  miR-324-5p  miR-5125  miR-335-3p  miR-344b-star  miR-500  miR-341-star  miR-135a-1-star  miR-871-5p  miR-183-star  miR-181c  miR-411-star  miR-467d-star  miR-129-2-3p  miR-215  miR-18a-star  miR-188-3p  miR-450b-5p  miR-3084  miR-361-star  miR-719  miR-1898  miR-5131  miR-125a-3p  miR-671-3p  miR-876-3p  miR-190b  miR-1943-star  miR-2861  miR-466e-3p  miR-99b  miR-423-5p  miR-467f  miR-133a-star  miR-669a-3p  miR-297c-star  miR-501-5p  miR-5109  miR-345-5p  miR-1948-star  miR-1945  miR-216a  miR-295  let-7e  let-7b  miR-3071  miR-292-5p  miR-3093-5p  miR-672  miR-25  miR-467c  miR-743a  miR-181b-1-star  miR-335-5p  miR-5113  miR-669d-2-star  miR-186-star  miR-203-star  miR-410-star  miR-300  miR-326  miR-874-star  miR-221  miR-712  miR-138  miR-144  miR-504  miR-1970  miR-200b  miR-764-3p  miR-687  miR-412-5p  miR-1941-5p  miR-3113-star  miR-466o-3p  miR-679-3p  miR-218  miR-1936  miR-652-star  miR-3090-star  miR-5108  miR-3113  miR-1963  miR-181b  miR-381  miR-694  miR-450b-3p  miR-3059  miR-466a-5p  miR-30d  miR-3110-star  miR-1843b-3p  miR-3103  miR-1930  miR-544-3p  miR-467a-star  miR-804  miR-3070a-star  miR-196b-star  miR-493-star  miR-669p  miR-3085-5p  miR-669a-3-3p  miR-491-star  miR-883b-5p  miR-130b-star  miR-299-star  miR-23a  miR-744-star  let-7g  miR-1927  miR-361  miR-181d-star  miR-532-3p  miR-145-star  miR-26a  miR-3068  miR-3092-star  miR-339-5p  miR-351-star  miR-152-star  miR-19a-star  miR-135b  miR-669h-5p  miR-3089-3p  miR-3097-5p  miR-3088-star  miR-290-5p  miR-96-star  miR-1298  miR-215-star  miR-218-1-star  miR-146a  miR-615-5p  miR-195-star  miR-101a-star  miR-154-star  miR-669g  let-7f-1-star  miR-5126  miR-133b-star  miR-294  miR-546  miR-329-star  miR-3106  miR-1934-star  miR-5130  miR-3074-5p  miR-3065-star  miR-324-3p  miR-296-5p  miR-874  miR-346-star  miR-667-star  miR-139-3p  miR-92a-2-star  miR-1a  miR-201  miR-29c-star  miR-3102-5p.2  miR-1954  miR-24-2-star  miR-148b-star  miR-224-star  miR-136-star  miR-132-star  miR-28  miR-449a-star  miR-450a  miR-1930-star  miR-129-5p  miR-539-5p  miR-337-5p  miR-676-star  miR-1198-3p  miR-205-star  miR-1947-star  miR-200b-star  miR-193b-star  miR-1981  miR-467g  miR-24  miR-511-3p  miR-3060  miR-665  miR-182  miR-3106-star  miR-129-1-3p  miR-92b  miR-7a-2-star  miR-29b-2-star  miR-3096-5p  miR-3966  miR-674-star  miR-294-star  miR-542-3p  miR-466a-3p  miR-3074-1-3p  miR-1968-star  miR-3112  miR-544-5p  miR-509-3p  miR-3057-5p  miR-1839-5p  miR-672-star  miR-3960  miR-29a  miR-3107-star  miR-3967  miR-378-star  miR-1193-3p  miR-490-3p  miR-466p-5p  miR-181d  miR-297b-3p  miR-3098-5p  miR-879  miR-188-5p  miR-184  miR-210-star  miR-615-3p  miR-138-2-star  miR-3096b-5p  miR-1983  miR-877  miR-698  miR-465b-5p  miR-379-star  miR-671-5p  let-7f  miR-149-star  miR-1942  miR-669d-star  miR-654-5p  miR-3067  miR-325  miR-466h-5p  miR-1247  miR-302b-star  miR-365-2-star  miR-700-star  miR-148b  miR-1928  miR-466b-3p  let-7d-star  miR-759  miR-5123  miR-199a-5p  miR-5102  miR-669m-5p  miR-598-star  miR-1193-5p  let-7c  miR-3087  miR-1251-star  miR-465a-3p  miR-200a-star  miR-342-3p  miR-149  miR-669n  miR-5135  miR-193  miR-706  miR-330  miR-466p-3p  miR-590-3p  miR-344c  miR-717  miR-675-3p  miR-1194  miR-448-5p  miR-23a-star  miR-302a  miR-412-3p  miR-770-3p  miR-1903  miR-5046  miR-802  miR-881-star  miR-409-5p  miR-1187  miR-150  miR-214-star  miR-99a  miR-203  miR-433-star  miR-1982-star  miR-486-star  miR-92b-star  miR-363-3p  miR-721  miR-582-3p  miR-669d  miR-222  miR-675-5p  miR-1906  miR-3962  miR-194  miR-1952  miR-466b-5p  miR-466m-5p  miR-488-star  miR-344c-star  miR-343  miR-470  miR-1935  miR-1931  miR-10b-star  miR-1934  miR-96  miR-194-1-star  miR-2136  miR-471-5p  miR-880  miR-125a-5p  miR-3069-5p  miR-505-5p  miR-137  miR-145  miR-155  miR-1955-3p  miR-30a  miR-493  miR-10b  miR-592-star  miR-1932  miR-297b-5p  miR-3074-2-3p  miR-26a-1-star  miR-1894-3p  miR-665-star  miR-3095-3p  miR-670-star  miR-374c  miR-1186b  miR-488  miR-344-star  miR-463  miR-666-5p  miR-761  miR-30e  miR-1b-5p  miR-3079-3p  miR-3077-star  miR-409-3p  miR-762  miR-22-star  miR-693-5p  miR-1197-star  miR-1958  miR-337-3p  miR-204  miR-3474  miR-132  miR-653  miR-3073-5p  miR-1940  miR-137-star  miR-320-star  miR-1224-star  miR-206-star  miR-669k-star  miR-5121  miR-125b-1-3p  miR-127-star  miR-5110  miR-202-5p  miR-425-star  miR-582-5p  miR-106a-star  miR-3089-5p  miR-16  miR-551b-star  miR-704  miR-466l-5p  miR-3095-5p  miR-764-5p  miR-351  miR-3971  miR-423-3p  miR-378b  miR-466e-5p  miR-466j  miR-682  miR-3080-3p  miR-466c-3p  miR-1199  miR-760-3p  miR-3094  miR-2182  miR-1948  miR-376a  miR-433  miR-299  miR-191-star  miR-16-1-star  miR-1981-star  miR-15a  miR-217  miR-547-star  miR-669e  let-7d  miR-494-star  miR-1a-1-star  miR-20b-star  miR-18a  miR-297c  miR-3057-3p  miR-1a-2-star  miR-3110  miR-598  miR-449a  miR-106b  miR-367  miR-466c-5p  miR-135a-2-star  miR-466o-5p  miR-5134  miR-1951  miR-466f  miR-668  miR-1933-5p  miR-191 | 1.8  1.6  1.4  1.1  1.4  1.4  1.0  -1.0  1.6  1.4  1.5  -1.3  1.6  1.3  1.3  1.7  -1.1  1.3  -1.2  -1.3  1.7  1.3  1.3  -1.0  1.2  1.3  -1.0  1.0  1.2  1.9  1.4  -1.1  1.3  1.3  1.6  9.4  1.3  2.8  1.1  -1.0  -1.2  1.1  -1.4  3.2  1.3  1.2  7.0  -1.3  1.2  -1.1  -1.0  1.7  1.5  -1.1  1.2  1.4  1.2  1.1  1.0  3.6  1.1  -1.2  1.1  1.4  1.4  1.3  1.1  -1.2  -1.1  -1.1  1.5  1.4  1.2  1.0  1.0  1.3  1.9  -1.2  -1.0  -1.1  1.0  -1.2  -1.2  1.7  1.4  2.3  -1.3  1.5  1.4  1.6  1.8  1.0  1.2  -1.1  1.5  -1.1  2.2  -1.5  -1.2  1.2  -1.1  -1.2  -1.1  1.8  1.3  1.4  -1.9  1.4  -1.0  -1.1  1.1  -1.0  1.1  1.7  -1.2  1.4  -1.2  -1.0  1.2  -1.2  1.2  -1.0  -1.1  1.3  -1.1  1.3  1.2  -1.1  -1.0  1.4  1.4  -1.3  1.2  1.3  1.1  -1.3  1.3  -1.2  1.9  1.1  -1.3  -1.3  -1.1  1.1  3.3  -1.2  3.2  1.0  1.3  -1.2  1.0  1.2  1.2  1.1  1.0  1.2  1.1  1.3  1.1  1.2  -1.1  -1.1  -1.4  1.3  1.1  1.1  1.1  -1.0  1.9  -1.2  -1.1  1.2  1.2  -1.0  1.9  -1.0  1.6  -2.6  1.2  2.4  -1.0  -1.2  -1.3  1.1  1.3  1.0  1.0  -1.0  -1.1  -1.0  -1.7  1.0  1.9  1.1  -1.2  1.3  1.4  1.4  1.2  1.0  1.2  1.0  1.2  1.3  1.2  -1.1  1.1  1.2  1.2  -1.4  1.4  -1.3  1.7  -1.2  -1.3  1.3  1.2  1.2  -1.0  1.0  -1.2  1.1  -1.2  -1.2  1.1  -1.1  1.4  1.0  1.7  1.1  -1.0  -1.2  -1.2  -1.2  1.3  -1.0  -1.1  -1.2  2.2  1.1  1.0  1.2  1.0  -1.1  1.1  -1.1  -1.0  1.8  -2.1  1.3  1.3  -1.2  -1.0  -1.2  1.1  1.0  -1.0  -1.1  1.2  -1.1  -1.2  1.1  1.2  -1.1  1.3  1.1  1.6  1.1  1.1  1.2  1.4  1.1  1.2  -1.0  -1.0  -1.1  1.2  -1.1  1.2  1.4  1.3  -1.1  -1.0  1.2  -1.0  -1.2  1.3  -1.3  1.4  2.3  1.2  2.0  2.0  1.0  1.1  1.4  1.1  -1.1  1.0  1.5  1.7  -1.1  -1.0  1.1  -1.1  1.1  1.1  -1.1  1.3  2.0  2.0  1.3  -1.1  -1.1  -1.0  -1.2  1.5  1.1  1.3  1.2  1.1  -1.3  1.0  1.3  -1.0  1.1  1.2  -1.2  1.1  -1.1  -1.0  -1.0  1.2  -1.2  -1.2  -1.1  1.1  1.1  1.1  1.0  1.2  -1.1  -1.4  1.6  -1.2  1.2  -1.1  -1.0  1.1  -1.1  1.1  1.1  1.6  -1.4  -1.1  -1.1  -1.2  -1.0  -1.1  -1.1  -1.2  -1.1  1.1  1.4  1.2  1.1  -1.1  1.7  1.2  1.3  -1.1  1.2  -1.0  -1.2  1.6  -1.0  1.3  -1.0  1.2  1.2  -1.2  -1.2  -1.2  1.3  -1.1  -1.1  1.0  -1.1  1.0  -1.0  1.1  -1.1  1.2  -1.0  1.1  1.2  1.2  -1.1  1.2  -1.1  -1.0  -1.0  1.1  -1.1  1.1  -1.1  1.2  1.0  -1.0  1.2  -1.2  -1.1  -1.0  1.1  -1.0  1.1  -1.1  1.2  1.3  -1.1  -1.2  1.2  1.3  1.0  1.1  1.3  1.0  1.1  -1.0  -1.0  -1.1  1.2  1.2  1.1  -1.0  -1.1  -1.1  -1.0  2.1  1.3  1.4  -1.1  1.2  -1.2  1.0  1.7  1.2  -1.1  -1.3  -1.0  1.2  1.1  1.1  -1.0  -1.1  1.1  1.1  1.1  1.1  1.0  1.2  -1.2  -1.1  1.0  -1.1  1.8  -1.0  -1.7  1.1  -1.1  -1.0  1.2  1.2  -1.1  -1.1  -1.2  1.2  1.0  1.2  1.4  1.4  -1.1  -1.1  -1.3  -1.1  -1.1  1.2  1.4  -1.1  1.1  -1.0  1.1  -1.0  1.1  1.0  1.2  1.8  -1.1  1.1  1.3  -1.2  1.3  1.6  -1.1  1.2  -1.6  1.1  1.1  -1.0  1.1  -1.1  1.1  -1.1  -1.0  1.1  -1.0  -1.0  1.1  1.2  1.2  1.1  -1.1  1.4  1.1  -1.1  1.0  1.1  1.0  -1.1  -1.2  -1.1  1.0  -1.1  -1.2  1.1  -1.1  1.1  -1.2  -1.1  -1.2  -1.2  1.5  1.0  1.1  1.0  1.0  -1.0  -1.1  1.0  1.0  -1.1  1.1  1.1  -1.1  -1.1  1.0  -1.1  -1.0  -1.0  -1.1  -1.0  1.0  1.4  -1.1  -1.1  -1.1  1.1  1.0  -1.0  1.8  1.0  -1.1  1.0  1.2  -1.1  1.2  -1.1  -1.0  1.0  1.2  -1.0  -1.1  1.0  -1.1  -1.2  -1.0  1.1  -1.1  1.1  1.0  -1.0  -1.1  -1.0  -1.0  1.1  1.1  1.0  -1.0  1.1  1.1  -1.1  -1.1  -1.1  -1.1  1.0  -1.3  1.1  -1.1  -1.1  -1.0  -1.2  1.0  1.1  1.2  1.0  1.1  -1.0  1.2  1.0  1.0  1.1  -1.0  1.5  1.1  -1.0  1.1  1.0  1.0  1.1  1.0  -1.2  -1.1  -1.1  -1.0  -1.0  -1.1  1.1  -1.1  -1.1  1.1  -1.1  -1.1  1.0  -1.6  1.2  1.0  1.1  1.4  -1.0  1.2  1.3  -1.1  1.1  1.1  1.6  -1.1  -1.2  1.1  1.0  1.1  -1.1  1.0  1.2  1.5  1.1  -1.0  1.1  -1.1  1.0  1.1  1.0  1.0  1.3  -1.1  1.0  -1.1  -1.1  -1.2  -1.1  1.0  1.0  1.3  -1.1  -1.1  -1.2  -1.0  1.0  -1.0  -1.1  -1.0  -1.0  1.0  1.1  1.1  -1.1  -1.1  -1.0  1.0  -1.1  1.3  -1.0  -1.0  -1.1  -1.1  1.1  -1.0  1.1  -1.0  1.1  1.1  1.6  1.1  -1.0  -1.0  -1.0  1.0  -1.1  1.0  1.0  -1.1  1.0  1.1  -1.0  1.1  1.3  -1.1  1.2  1.2  1.1  -1.1  1.0  1.3  1.0  -1.1  -1.0  1.0  1.0  1.1  1.1  -1.0  -1.0  1.0  -1.0  -1.1  -1.1  -1.1  1.1  -1.3  -1.1  -1.1  1.1  1.0  -1.1  -1.1  1.0  -1.0  1.1  1.1  1.2  1.1  1.1  -1.1  1.1  1.1  1.0  -1.1  1.0  -1.1  1.1  1.1  1.0  1.1  1.1  1.4  -1.1  -1.0  1.1  -1.0  -1.0  1.0  -1.0  -1.1  1.0  1.0  -1.1  1.1  1.1  -1.0  -1.0  -1.0  1.0  -1.0  -1.0  -1.0  1.0  -1.0  -1.0  -1.0  1.1  -1.0  1.1  1.1  1.0  -1.1  1.0  1.0  -1.0  -1.0  -1.0  -1.0  -1.1  -1.1  -1.1  1.1  1.1  -1.0  -1.0  -1.1  -1.0  1.0  -1.1  1.1  -1.0  -1.1  -1.0  -1.0  1.0  -1.0  1.0  -1.0  1.0  -1.0  1.0  1.1  1.1  -1.1  1.1  -1.0  -1.0  -1.0  1.1  1.0  1.0  1.1  1.0  -1.1  1.0  1.1  1.0  -1.1  -1.0  1.1  1.2  -1.0  -1.1  1.0  1.0  -1.0  1.0  -1.0  1.0  -1.0  1.0  -1.1  1.0  1.0  1.0  1.1  -1.0  1.0  -1.0  -1.0  1.0  -1.0  -1.0  1.0  1.0  -1.0  -1.0  1.0  -1.0  1.0  1.0  1.0  1.0  1.0  1.0  -1.0  1.0  1.0 | 0.0000  0.0002  0.0010  0.4215  0.0038  0.1742  0.8617  0.8724  0.0011  0.0303  0.0305  0.0669  0.0006  0.0166  0.0036  0.0041  0.0401  0.0021  0.1048  0.0064  0.0022  0.0080  0.0067  0.9496  0.3372  0.0155  0.7227  0.9214  0.0505  0.0033  0.0102  0.5787  0.1522  0.0693  0.0261  0.0022  0.0296  0.0013  0.6356  0.9386  0.0082  0.8094  0.1490  0.0057  0.0879  0.0463  0.0018  0.0038  0.0039  0.0451  0.5225  0.0050  0.0064  0.5523  0.0444  0.0110  0.0439  0.4014  0.9110  0.0048  0.1139  0.0079  0.5502  0.0099  0.0496  0.0191  0.5832  0.0347  0.5572  0.2978  0.0390  0.2611  0.5090  0.6950  0.8552  0.0301  0.0384  0.0617  0.7808  0.1255  0.8287  0.0089  0.1027  0.0182  0.0095  0.0482  0.0093  0.1295  0.0975  0.0159  0.0059  0.9431  0.0248  0.6880  0.0083  0.1534  0.0910  0.0520  0.0432  0.2492  0.2500  0.1421  0.2808  0.1674  0.0112  0.1090  0.0222  0.0077  0.6924  0.6643  0.4679  0.6582  0.2429  0.0309  0.1590  0.0128  0.0081  0.9385  0.0223  0.0680  0.0941  0.6825  0.2221  0.1042  0.1558  0.0825  0.0097  0.2272  0.9200  0.1878  0.0968  0.0392  0.0297  0.0242  0.2980  0.0114  0.0157  0.0484  0.0324  0.1102  0.0181  0.0106  0.0324  0.5128  0.0170  0.0232  0.0257  0.9577  0.0158  0.1066  0.6655  0.0266  0.0203  0.0973  0.8840  0.0314  0.0948  0.0199  0.5995  0.0479  0.2996  0.3812  0.2044  0.0244  0.1767  0.5461  0.1877  0.9485  0.1833  0.0391  0.0748  0.1766  0.1885  0.8824  0.0261  0.7005  0.1923  0.0473  0.2038  0.0285  0.6693  0.0247  0.0316  0.1008  0.0385  0.9482  0.8266  0.8218  0.7745  0.9390  0.0876  0.8887  0.0468  0.0800  0.0267  0.0425  0.0218  0.3736  0.0657  0.7846  0.1732  0.6691  0.4633  0.4702  0.1641  0.6922  0.6805  0.0446  0.0694  0.3005  0.0307  0.0276  0.0427  0.0491  0.1817  0.1155  0.1691  0.0533  0.8679  0.9690  0.1846  0.3025  0.0857  0.3140  0.5391  0.4815  0.3704  0.7137  0.2011  0.4734  0.9390  0.0676  0.0497  0.0662  0.0734  0.6423  0.2053  0.0363  0.0576  0.1440  0.7594  0.1401  0.7420  0.5160  0.1292  0.2964  0.6152  0.0571  0.0664  0.3749  0.1419  0.2568  0.8794  0.0781  0.5645  0.8129  0.8729  0.4622  0.1954  0.3294  0.0777  0.5713  0.0549  0.3375  0.0351  0.4133  0.0769  0.2504  0.5882  0.2543  0.2902  0.4664  0.0693  0.9865  0.8729  0.2654  0.0693  0.7002  0.1775  0.0360  0.0834  0.1743  0.9450  0.3800  0.7300  0.1079  0.3826  0.0633  0.0940  0.0664  0.1544  0.1419  0.0740  0.8577  0.5761  0.2691  0.1610  0.3705  0.6371  0.1982  0.0491  0.3550  0.6574  0.5500  0.4663  0.2802  0.4356  0.5226  0.0637  0.0442  0.0707  0.0922  0.1247  0.4070  0.8217  0.0986  0.0437  0.4295  0.2707  0.1377  0.7515  0.1203  0.7687  0.3112  0.6935  0.2284  0.3442  0.1048  0.3755  0.2285  0.8999  0.9331  0.2214  0.0696  0.1040  0.7228  0.3735  0.1512  0.1977  0.6003  0.1457  0.4822  0.3746  0.2094  0.0597  0.1232  0.5190  0.7626  0.4695  0.3377  0.3377  0.7016  0.0819  0.2604  0.1527  0.1901  0.0736  0.9993  0.3261  0.4649  0.1145  0.4204  0.4317  0.4946  0.0673  0.5363  0.0815  0.1568  0.4974  0.0797  0.3624  0.0689  0.9590  0.1426  0.1017  0.8715  0.0694  0.8011  0.1282  0.0802  0.1315  0.1667  0.1073  0.1717  0.4900  0.5503  0.7700  0.3844  0.9659  0.6913  0.1571  0.3937  0.0832  0.7067  0.1702  0.2381  0.2627  0.2059  0.0980  0.2423  0.9422  0.6131  0.3888  0.6872  0.4471  0.2396  0.2369  0.9007  0.8707  0.2283  0.1643  0.1795  0.6890  0.2297  0.8294  0.3242  0.3052  0.2321  0.1030  0.2605  0.1235  0.1662  0.2187  0.7357  0.5516  0.3454  0.6648  0.3974  0.7207  0.6635  0.5009  0.2158  0.2264  0.2266  0.8091  0.2199  0.4127  0.5188  0.1186  0.1538  0.1404  0.5933  0.2187  0.1327  0.6442  0.1124  0.1354  0.1208  0.1215  0.9149  0.1692  0.3671  0.5309  0.7134  0.4888  0.5241  0.5688  0.2178  0.2321  0.8133  0.1372  0.1021  0.3594  0.8429  0.6264  0.1578  0.7578  0.2443  0.5273  0.4947  0.7297  0.1946  0.1521  0.1334  0.1831  0.1814  0.2772  0.7685  0.6379  0.1311  0.1357  0.4605  0.3286  0.1533  0.6546  0.1599  0.3760  0.4862  0.2590  0.5399  0.9695  0.3635  0.7070  0.6251  0.5760  0.1855  0.1606  0.2926  0.2093  0.1936  0.5400  0.3747  0.1251  0.3162  0.2585  0.1251  0.2295  0.3188  0.9705  0.4554  0.2155  0.4307  0.2903  0.6158  0.3191  0.9290  0.6454  0.2935  0.3357  0.1307  0.3571  0.2649  0.1446  0.7031  0.1562  0.8136  0.2197  0.8785  0.2227  0.1426  0.5839  0.6538  0.2439  0.1955  0.2411  0.2940  0.7321  0.2740  0.2318  0.2531  0.2485  0.1577  0.6967  0.2434  0.8431  0.6340  0.8238  0.4586  0.8602  0.9525  0.5058  0.2642  0.2560  0.1888  0.2806  0.7386  0.2997  0.9019  0.7467  0.6306  0.6460  0.9356  0.2120  0.2364  0.5576  0.1710  0.3966  0.8023  0.9736  0.1612  0.8019  0.2696  0.9284  0.4526  0.5308  0.6561  0.1832  0.7895  0.8690  0.3060  0.8763  0.3911  0.5282  0.2799  0.1979  0.9697  0.4731  0.5152  0.1754  0.8359  0.5621  0.4145  0.7273  0.8479  0.7166  0.3153  0.6279  0.8086  0.2038  0.2446  0.2739  0.3319  0.5661  0.3767  0.6299  0.3298  0.5685  0.6902  0.5198  0.9536  0.1740  0.9634  0.3698  0.2521  0.8405  0.2491  0.7575  0.1821  0.8151  0.7686  0.4852  0.9458  0.2692  0.2617  0.8903  0.5488  0.8111  0.9006  0.4054  0.7289  0.2066  0.6350  0.5415  0.9314  0.7069  0.2239  0.3391  0.1990  0.2341  0.5268  0.2734  0.2763  0.8454  0.2258  0.4649  0.7273  0.3639  0.2985  0.7292  0.4923  0.2985  0.3198  0.6955  0.2342  0.2150  0.7479  0.2658  0.2811  0.9585  0.3429  0.6293  0.8306  0.5066  0.2830  0.2738  0.8652  0.4399  0.4548  0.7024  0.4278  0.9863  0.9110  0.5914  0.4102  0.7820  0.3613  0.3403  0.3395  0.2277  0.7257  0.9139  0.3535  0.2786  0.2454  0.3821  0.7061  0.9787  0.6312  0.3013  0.8796  0.6910  0.9773  0.6095  0.4858  0.4598  0.3171  0.5175  0.7640  0.4144  0.2589  0.8878  0.9242  0.4549  0.7372  0.5463  0.7925  0.2575  0.7851  0.5539  0.5491  0.3471  0.4160  0.8038  0.5633  0.7372  0.9981  0.7292  0.7382  0.9328  0.5132  0.7446  0.3168  0.4843  0.2818  0.3172  0.7586  0.2851  0.3295  0.5056  0.3124  0.6466  0.4961  0.6499  0.4126  0.7435  0.5908  0.8348  0.5526  0.4099  0.6382  0.6244  0.8445  0.8170  0.5425  0.7611  0.5963  0.3304  0.4383  0.5782  0.3569  0.3767  0.8995  0.3585  0.4937  0.8416  0.7774  0.3951  0.4590  0.6799  0.5745  0.7442  0.3540  0.4513  0.4698  0.9628  0.5477  0.8458  0.7666  0.4313  0.6226  0.8526  0.3148  0.6920  0.3511  0.5304  0.6953  0.5188  0.9952  0.5033  0.8480  0.7820  0.6321  0.9684  0.7320  0.4524  0.5458  0.6814  0.8485  0.9739  0.5463  0.6197  0.7573  0.5361  0.7342  0.8791  0.6754  0.5740  0.9687  0.3978  0.8017  0.6194  0.4303  0.7017  0.5405  0.8193  0.8372  0.9743  0.7867  0.6927  0.8096  0.6269  0.4704  0.4977  0.6713  0.4895  0.6780  0.9047  0.4766  0.6564  0.7852  0.5421  0.4762  0.9256  0.6354  0.7464  0.9579  0.8675  0.8078  0.6740  0.9558  0.6819  0.7872  0.8602  0.8354  0.5703  0.5801  0.6191  0.8914  0.9979  0.7594  0.6344  0.9432  0.8281  0.4899  0.6935  0.5784  0.7006  0.6666  0.7249  0.7251  0.6606  0.6493  0.6098  0.7580  0.5079  0.6873  0.7509  0.7630  0.8047  0.8624  0.9011  0.9372  0.7728  0.6788  0.6460  0.8720  0.9932  0.6322  0.8113  0.8193  0.8961  0.9303  0.6704  0.9202  0.9624  0.6307  0.9812  0.9442  0.7571  0.7710  0.8809  0.7040  0.9267  0.8429  0.9047  0.8852  0.8696  0.9251  0.8598  0.9711 | 1.3  1.2  1.0  1.0  -1.0  -1.9  1.2  1.2  1.1  -1.2  -1.2  -1.6  1.3  1.5  -1.0  1.5  1.0  1.1  -1.1  -1.3  1.7  1.3  -1.0  -1.7  -1.5  -1.1  -1.3  2.0  1.2  1.1  1.1  -1.5  1.8  1.2  -1.1  2.1  1.4  2.0  -1.7  -1.6  -1.2  2.3  -1.9  3.5  1.3  1.2  2.7  -1.3  1.2  1.0  1.2  1.2  1.1  -1.3  -1.1  1.0  1.1  1.2  -1.5  1.5  1.2  -1.2  1.3  1.1  -1.1  1.0  1.3  1.0  1.2  -1.2  -1.1  -1.6  -1.6  1.4  1.3  1.4  2.5  -1.2  -1.6  -1.1  1.3  -1.1  1.0  1.8  1.3  2.6  -1.3  -1.4  -1.0  1.1  1.4  -1.3  -1.0  1.4  1.3  -1.0  2.9  -1.5  -1.3  -1.3  -1.2  1.1  -1.1  -1.6  1.1  -1.1  -1.2  1.2  -1.2  1.5  -1.2  1.2  1.1  1.9  -1.5  1.1  -1.1  1.3  1.2  1.1  -1.1  1.0  1.1  -1.1  -1.2  1.0  1.2  -1.2  1.1  1.6  1.1  -1.4  1.0  1.3  1.3  -1.1  1.1  1.0  1.2  1.2  -1.1  -1.2  -1.1  -1.3  1.6  -1.1  1.4  1.2  1.1  -1.1  -1.2  1.0  1.1  -1.0  2.0  1.2  -1.0  1.2  1.2  1.0  -1.1  1.1  -1.5  1.1  -1.0  1.2  1.3  1.7  -1.2  -1.2  -1.0  -1.1  1.4  1.1  1.2  -1.0  2.0  -1.1  -1.0  2.3  -1.0  -1.1  -1.1  1.0  1.1  1.7  1.2  1.2  -1.5  1.2  -2.1  1.3  1.2  1.1  -1.1  1.0  1.2  -1.7  1.0  1.2  1.3  1.2  -1.3  2.2  -1.1  1.6  -1.5  1.2  -1.0  1.4  1.1  -1.2  1.3  -1.2  1.1  -1.1  1.0  1.0  1.1  -1.1  -1.1  1.2  -1.1  1.1  -1.2  1.2  -1.5  -1.1  -1.3  -1.2  1.1  -1.2  -1.1  -1.2  -1.0  1.1  -1.0  -1.1  1.1  1.0  1.2  1.2  1.2  1.2  1.1  -1.0  -1.2  1.6  -2.3  -1.4  -1.1  1.1  -1.5  -1.2  1.3  1.0  -1.1  -1.2  1.1  -1.1  -1.1  1.2  1.2  1.0  1.2  1.2  1.2  -1.1  1.4  -1.2  -1.4  1.0  1.0  -1.1  1.1  -1.2  1.0  -1.0  1.0  1.2  1.0  -1.1  1.1  1.6  1.2  -1.2  1.5  -1.2  1.3  2.4  1.0  2.1  2.0  1.0  1.3  1.8  1.1  1.1  1.2  2.0  1.6  -1.0  1.1  -1.1  1.1  1.1  -1.1  1.1  1.1  1.5  2.0  1.0  1.0  -1.2  1.1  -1.0  1.3  1.3  -1.0  -1.0  1.3  1.0  1.1  -1.1  -1.1  -1.0  -1.1  -1.2  -1.1  -1.2  1.2  1.1  -1.0  -1.0  -1.1  -1.5  -1.1  1.1  1.1  1.1  1.1  -1.2  1.1  -1.2  -1.1  -1.0  1.1  -1.1  1.1  1.0  1.1  1.4  1.6  -1.5  -1.2  1.0  -1.1  1.1  -1.1  1.1  -1.1  1.1  1.2  -1.1  1.1  1.2  -1.0  1.9  1.6  1.2  -1.1  1.1  1.0  -1.0  1.5  1.0  1.2  -1.2  1.1  1.1  -1.2  -1.2  -1.0  -1.0  -1.1  -1.0  1.2  1.0  1.1  -1.2  1.0  1.0  1.2  1.1  1.1  1.2  1.2  -1.1  1.1  -1.0  -1.1  1.1  1.2  1.2  1.2  -1.1  -1.1  1.2  1.1  1.3  -1.2  -1.1  1.1  1.0  1.1  1.1  -1.0  1.3  1.2  1.0  -1.1  1.2  1.3  -1.0  1.1  1.0  1.1  1.2  1.0  1.1  -1.1  1.2  -1.0  1.1  1.2  -1.1  1.0  1.0  1.9  1.3  1.1  -1.1  -1.0  -1.1  1.1  1.5  1.0  -1.1  -1.2  1.4  1.1  1.2  1.1  -1.1  1.1  1.3  1.1  1.2  1.1  1.1  1.0  -1.1  -1.1  -1.1  -1.2  1.8  1.1  1.0  -1.2  -1.1  1.1  1.1  1.2  -1.1  -1.1  -1.0  -1.1  1.1  1.5  1.3  1.4  1.0  -1.1  -1.3  -1.4  -1.0  -1.1  -1.4  -1.1  -1.1  -1.1  1.1  -1.1  1.2  1.1  1.2  1.3  -1.0  1.2  1.4  -1.6  1.3  1.3  1.0  1.0  -1.4  1.2  -1.0  -1.2  1.2  -1.1  1.0  -1.0  1.1  1.1  1.1  1.0  1.1  -1.0  1.2  -1.1  -1.0  1.3  1.2  -1.1  1.1  1.1  1.1  -1.1  -1.1  1.0  1.1  1.0  -1.2  1.1  1.0  -1.2  -1.1  -1.1  -1.1  -1.0  1.2  1.1  -1.0  1.2  1.1  -1.1  1.0  1.2  -1.1  1.0  1.0  1.0  -1.0  -1.1  -1.1  1.0  -1.2  1.1  1.0  -1.1  -1.0  1.4  -1.1  -1.0  -1.1  1.2  1.1  1.2  1.5  -1.0  -1.0  1.1  1.3  -1.1  1.6  -1.1  -1.1  1.1  -1.0  -1.1  -1.2  -1.0  -1.0  -1.2  1.1  1.0  1.0  1.1  -1.1  -1.1  -1.1  1.1  -1.1  -1.0  1.1  1.1  1.1  1.0  1.0  1.0  -1.2  -1.2  -1.1  1.1  1.1  -1.1  1.0  -1.2  -1.2  -1.1  1.0  1.1  1.3  1.2  1.0  1.1  1.1  1.1  1.1  1.1  -1.1  1.5  1.1  -1.3  1.1  1.0  -1.0  1.0  1.1  -1.2  -1.1  1.0  1.1  1.0  -1.0  1.2  -1.1  -1.0  1.1  -1.1  -1.1  1.1  -1.2  1.0  1.0  1.1  1.1  1.1  -1.1  1.3  -1.1  1.3  1.1  1.4  1.1  -1.2  1.0  -1.1  -1.0  -1.2  -1.1  -1.1  1.5  1.1  1.1  1.0  -1.1  -1.0  1.1  1.1  -1.1  -1.2  -1.0  -1.1  -1.2  -1.0  1.0  -1.0  -1.1  1.1  1.0  -1.1  -1.1  1.0  -1.1  1.0  -1.1  -1.1  1.1  -1.1  1.1  1.1  1.1  1.0  -1.0  1.0  -1.1  -1.0  1.2  -1.1  -1.1  -1.0  -1.3  1.0  1.0  1.1  -1.0  -1.0  1.0  1.7  1.1  -1.1  -1.1  -1.0  1.0  -1.2  -1.0  1.0  1.1  1.1  1.1  -1.0  1.1  1.2  1.1  1.1  1.0  1.1  -1.1  1.1  1.1  -1.0  1.0  -1.0  1.0  -1.0  1.1  1.1  -1.1  1.1  1.1  -1.1  1.1  -1.0  -1.1  1.1  1.0  -1.0  -1.1  1.1  1.1  -1.0  1.0  -1.0  1.1  1.1  -1.0  1.4  -1.0  -1.1  -1.0  1.2  1.0  1.0  1.0  -1.1  1.1  -1.0  1.1  -1.0  1.1  -1.0  1.4  1.0  -1.0  -1.0  -1.1  -1.1  1.0  1.0  -1.1  1.1  -1.0  -1.1  1.1  1.1  -1.0  1.0  1.0  1.1  -1.0  1.0  -1.1  -1.0  -1.1  1.0  1.1  1.1  1.0  1.1  1.0  1.1  -1.0  -1.0  -1.0  -1.1  -1.0  -1.0  -1.1  -1.1  -1.0  -1.0  1.1  1.0  -1.0  -1.1  -1.1  -1.0  1.1  -1.0  1.1  1.0  -1.1  1.0  1.0  -1.1  -1.0  1.1  1.1  1.0  -1.1  -1.0  -1.1  1.0  1.0  -1.0  -1.0  1.1  1.0  1.1  -1.0  1.1  1.0  1.1  1.0  1.0  1.1  1.1  -1.0  -1.0  -1.0  1.1  1.0  -1.0  1.0  1.1  -1.1  -1.0  -1.0  1.0  1.0  -1.0  -1.0  1.0  1.0  -1.0  1.1  -1.1  1.0  -1.0  1.0  1.0  1.0  -1.0  1.0  -1.0  -1.0  -1.0  1.0  -1.0  1.0  -1.0  -1.0  1.0  1.0  1.0  1.0  1.0  -1.0 | 0.0103  0.1261  0.8943  0.5595  0.6378  0.0171  0.0211  0.0064  0.4228  0.2797  0.2371  0.0029  0.0446  0.0004  0.9735  0.0122  0.4302  0.1494  0.4913  0.0017  0.0019  0.0126  0.7488  0.0054  0.0516  0.4434  0.0224  0.0165  0.0565  0.6400  0.6568  0.0026  0.0065  0.0893  0.5272  0.2651  0.0051  0.0223  0.0296  0.0094  0.0439  0.0153  0.0059  0.0034  0.0439  0.0130  0.0826  0.0057  0.0411  0.3601  0.0453  0.3380  0.6681  0.0089  0.4670  0.8988  0.4626  0.0174  0.0158  0.2988  0.0037  0.0077  0.0698  0.5794  0.5466  0.9543  0.0253  0.7539  0.0456  0.0461  0.6119  0.1087  0.0799  0.0126  0.0136  0.0052  0.0044  0.0153  0.0129  0.2011  0.0468  0.2195  0.8725  0.0105  0.0625  0.0252  0.0139  0.2436  0.9578  0.4883  0.0872  0.0574  0.9728  0.0588  0.0717  0.7515  0.0255  0.0270  0.0070  0.1794  0.0500  0.2653  0.4363  0.2292  0.4067  0.4824  0.5467  0.1237  0.1103  0.0591  0.1299  0.0944  0.3983  0.0124  0.0096  0.4180  0.1139  0.0330  0.0200  0.5594  0.4468  0.7615  0.3571  0.4022  0.0105  0.9691  0.0460  0.0193  0.0916  0.0460  0.5521  0.0152  0.8940  0.0158  0.0268  0.2392  0.2612  0.8745  0.4530  0.0135  0.4451  0.1186  0.0142  0.0957  0.3405  0.0486  0.4939  0.0498  0.3582  0.5662  0.0750  0.6961  0.2670  0.5411  0.0344  0.0293  0.7498  0.1941  0.0755  0.8314  0.2103  0.3056  0.0841  0.5950  0.7710  0.0574  0.0183  0.0543  0.6618  0.0416  0.7923  0.5560  0.0173  0.2817  0.4945  0.6826  0.0534  0.9065  0.6853  0.0394  0.8764  0.3062  0.4953  0.7232  0.4945  0.0516  0.0881  0.1867  0.0812  0.0814  0.0204  0.0813  0.5789  0.0875  0.0562  0.7867  0.2169  0.1992  0.7066  0.1249  0.0330  0.0414  0.1733  0.0372  0.6854  0.1058  0.1099  0.0537  0.9420  0.2678  0.3460  0.0838  0.2914  0.0443  0.4777  0.7667  0.8664  0.7691  0.1565  0.1716  0.5139  0.0396  0.6183  0.7050  0.3429  0.1710  0.2530  0.2483  0.5414  0.1982  0.0748  0.0330  0.2944  0.0773  0.9774  0.1876  0.9577  0.1895  0.7632  0.9262  0.0750  0.1694  0.0780  0.1713  0.4013  0.7629  0.0487  0.1143  0.0398  0.2859  0.6292  0.4073  0.0653  0.0971  0.1573  0.6195  0.0684  0.0398  0.3704  0.4990  0.5445  0.0785  0.0644  0.9117  0.1505  0.0638  0.5388  0.4111  0.0509  0.4403  0.3507  0.9301  0.8421  0.3707  0.3516  0.0392  0.8279  0.8070  0.8187  0.2234  0.8558  0.2614  0.2483  0.0453  0.1319  0.1494  0.1270  0.1134  0.1394  0.0547  0.6755  0.1183  0.0674  0.9186  0.1263  0.0479  0.1274  0.3760  0.1292  0.0429  0.0942  0.7649  0.2363  0.4662  0.3838  0.1148  0.4380  0.5113  0.3833  0.2036  0.0658  0.9480  0.8830  0.2888  0.3991  0.9425  0.2370  0.0701  0.9389  0.9821  0.0792  0.9752  0.1458  0.6084  0.1889  0.7647  0.6611  0.0660  0.5147  0.0645  0.1490  0.2214  0.8807  0.5953  0.1796  0.0769  0.3510  0.4963  0.1655  0.2166  0.7054  0.0614  0.7396  0.6279  0.3561  0.9826  0.4212  0.2831  0.3898  0.5327  0.2009  0.0840  0.1112  0.1375  0.0667  0.7135  0.3585  0.1439  0.5609  0.3156  0.4808  0.3537  0.1473  0.8257  0.1882  0.0766  0.5903  0.0779  0.0917  0.1193  0.2027  0.2589  0.8817  0.9012  0.1125  0.7853  0.2989  0.1599  0.5256  0.1943  0.1730  0.1376  0.7878  0.9748  0.3407  0.8848  0.1060  0.8204  0.5794  0.2150  0.9179  0.7595  0.1422  0.2854  0.3713  0.1177  0.2559  0.0852  0.5494  0.4799  0.3183  0.3482  0.1552  0.2396  0.2687  0.2765  0.6998  0.1352  0.2319  0.1315  0.1962  0.1239  0.2801  0.8663  0.2896  0.1203  0.5699  0.1031  0.1440  0.7567  0.3968  0.1068  0.2387  0.9263  0.3447  0.9882  0.1785  0.1594  0.8357  0.3249  0.4055  0.0917  0.8356  0.2428  0.2359  0.0984  0.5477  0.5701  0.1760  0.1809  0.7252  0.5519  0.9563  0.2605  0.2005  0.1901  0.6292  0.2411  0.2063  0.2292  0.4236  0.0990  0.1343  0.1837  0.4248  0.1112  0.5736  0.1437  0.4819  0.2060  0.7509  0.2549  0.3119  0.3969  0.1383  0.1507  0.4766  0.9288  0.3883  0.3552  0.2934  0.3889  0.1448  0.1563  0.4074  0.9309  0.7615  0.3768  0.1839  0.1896  0.1656  0.7847  0.3793  0.1908  0.1780  0.8355  0.7280  0.4944  0.5437  0.4737  0.4092  0.5409  0.6283  0.1715  0.1497  0.1397  0.4980  0.9603  0.1307  0.1373  0.2070  0.3592  0.4341  0.9070  0.8941  0.2663  0.1341  0.8208  0.2532  0.1774  0.2632  0.8491  0.9070  0.3928  0.3499  0.3284  0.4988  0.1323  0.8326  0.2792  0.6523  0.8109  0.2549  0.1738  0.4898  0.2963  0.4230  0.2881  0.2389  0.2494  0.8059  0.2437  0.9280  0.3371  0.1456  0.8097  0.3413  0.4746  0.1841  0.3900  0.8487  0.4643  0.4747  0.9480  0.2122  0.1667  0.1901  0.7117  0.2201  0.5236  0.6891  0.9986  0.9766  0.8109  0.1647  0.3842  0.8329  0.2112  0.3905  0.6879  0.1900  0.8591  0.2274  0.2259  0.8141  0.4016  0.1498  0.2419  0.2508  0.3236  0.4760  0.6164  0.5499  0.2687  0.4739  0.2269  0.2207  0.2933  0.3578  0.8734  0.7128  0.1654  0.5653  0.9795  0.2324  0.3188  0.7587  0.8919  0.3414  0.3169  0.2582  0.1875  0.3914  0.3205  0.8935  0.4278  0.2162  0.3920  0.5521  0.8701  0.9965  0.1959  0.2047  0.5825  0.3525  0.8328  0.5352  0.8928  0.1781  0.2594  0.4615  0.6919  0.2146  0.2479  0.2353  0.6671  0.3817  0.5211  0.4471  0.2686  0.3173  0.3789  0.2154  0.3113  0.2595  0.3704  0.8612  0.7403  0.8010  0.5262  0.3443  0.2488  0.8366  0.3284  0.9970  0.7618  0.1906  0.3419  0.4959  0.3090  0.4886  0.3647  0.2509  0.6095  0.8868  0.7207  0.5122  0.8290  0.4536  0.6251  0.3177  0.3027  0.2463  0.5252  0.3299  0.6614  0.2721  0.7198  0.4366  0.9216  0.2312  0.3844  0.6688  0.2877  0.3849  0.3883  0.8154  0.2572  0.7885  0.3625  0.3618  0.4454  0.7614  0.7616  0.5890  0.2371  0.9888  0.9996  0.4898  0.4689  0.4306  0.8933  0.3406  0.6394  0.8980  0.3524  0.8701  0.3069  0.2737  0.4046  0.3891  0.4571  0.5420  0.4391  0.8909  0.8779  0.8590  0.5761  0.8292  0.3650  0.4511  0.3878  0.6306  0.2906  0.6939  0.6435  0.5159  0.8816  0.7624  0.5928  0.3031  0.3067  0.3672  0.3227  0.9900  0.9414  0.3681  0.9457  0.7635  0.7493  0.2977  0.3158  0.5569  0.3693  0.4908  0.5857  0.3774  0.8585  0.3057  0.5495  0.4518  0.8669  0.6305  0.9914  0.8441  0.6941  0.6980  0.2782  0.3571  0.2935  0.6219  0.3908  0.3696  0.7367  0.8848  0.4161  0.4344  0.9016  0.8030  0.3376  0.3949  0.5996  0.8847  0.8152  0.8924  0.6072  0.4562  0.8850  0.3217  0.8926  0.6498  0.8295  0.3106  0.7384  0.5641  0.7733  0.4971  0.6869  0.9750  0.3804  0.5632  0.5259  0.8391  0.4089  0.8026  0.6759  0.9636  0.4395  0.3490  0.6878  0.5642  0.4250  0.5023  0.6091  0.3893  0.4245  0.3507  0.8043  0.9480  0.8240  0.5007  0.6507  0.8765  0.4914  0.6740  0.5325  0.9970  0.4845  0.4436  0.6602  0.6839  0.9003  0.4338  0.8111  0.9383  0.8998  0.4768  0.6491  0.9264  0.6665  0.4955  0.9637  0.8027  0.3963  0.9951  0.6698  0.5749  0.4418  0.6108  0.5850  0.6927  0.6717  0.6806  0.6032  0.7014  0.6349  0.6132  0.4688  0.5943  0.5838  0.7383  0.4944  0.9556  0.7192  0.9910  0.9521  0.9104  0.6015  0.5517  0.7248  0.4939  0.9741  0.5458  0.7829  0.6076  0.9567  0.8379  0.4756  0.4945  0.8895  0.7850  0.8903  0.7364  0.8191  0.6697  0.7101  0.5325  0.5306  0.8882  0.9114  0.6825  0.7298  0.9064  0.9926  0.8409  0.6022  0.8067  0.5811  0.6274  0.8390  0.7713  0.7387  0.7310  0.7259  0.7685  0.7933  0.7688  0.9532  0.6647  0.6774  0.7998  0.7850  0.8867  0.9428  0.9550  0.8040  0.8647  0.9432  0.9120  0.9583 | 1.1  1.1  1.0  -1.3  -1.0  -1.5  1.3  -1.0  1.4  -1.2  -1.2  -1.7  1.2  1.3  1.0  1.9  -1.1  1.0  1.2  -1.3  1.6  1.4  1.1  -1.4  -1.5  1.0  -1.3  2.1  1.4  1.1  -1.1  -1.2  1.9  1.6  -1.1  2.1  1.1  1.9  -1.5  -1.4  -1.3  2.2  -1.9  3.1  -1.1  1.3  2.6  -1.3  1.1  -1.0  -1.0  1.1  1.2  -1.0  -1.0  1.2  -1.1  1.3  -1.1  1.7  1.1  -1.2  1.6  1.1  -1.1  1.2  1.3  1.0  1.1  1.1  -1.0  -1.3  -1.5  1.1  1.1  1.3  1.7  -1.0  -1.2  1.1  -1.1  -1.2  -1.2  1.3  1.1  3.3  -1.2  1.1  -1.2  1.1  1.4  -1.3  1.1  -1.0  1.2  -1.3  3.6  -1.7  -1.2  -1.1  1.0  1.0  1.1  -1.1  1.1  -1.1  -1.8  1.2  1.1  -1.0  -1.2  1.2  1.2  1.4  -1.2  1.1  -1.1  1.1  1.2  -1.1  -1.0  1.1  -1.1  -1.0  -1.2  -1.1  1.1  -1.2  -1.0  1.9  -1.2  -1.2  1.0  1.2  1.3  -1.1  1.1  -1.0  1.0  1.2  -1.2  -1.2  -1.1  -1.1  1.5  -1.1  1.2  1.0  1.1  -1.4  -1.1  1.1  1.0  1.0  1.3  1.1  1.1  1.1  -1.0  1.0  -1.3  1.1  -1.8  1.1  1.1  1.2  1.1  1.1  -1.5  -1.2  1.0  -1.1  1.3  1.1  1.2  1.1  1.0  -1.2  1.2  2.2  1.2  -1.2  -1.2  1.2  1.3  1.3  1.2  -1.1  -1.6  -1.0  -1.6  1.2  1.1  1.0  -1.1  1.1  1.2  -1.1  -1.0  1.2  1.1  1.1  -1.1  1.8  -1.1  1.2  -1.2  1.1  1.1  -1.0  1.2  -1.1  1.1  -1.2  -1.1  -1.0  -1.1  1.0  1.1  -1.2  1.1  1.0  -1.3  -1.3  -1.3  1.0  -1.2  1.1  -1.2  1.0  1.0  -1.2  -1.0  -1.2  1.1  -1.0  1.1  -1.1  1.2  1.1  1.2  1.3  1.2  1.0  1.2  -1.1  -1.1  1.9  -1.7  -1.2  1.0  1.0  -1.2  -1.0  -1.1  1.2  -1.0  -1.1  1.3  1.1  -1.0  1.0  1.1  -1.2  1.1  1.2  1.0  -1.0  1.2  -1.1  1.0  1.3  1.1  1.1  -1.1  -1.1  1.1  1.3  1.2  1.2  1.2  1.0  -1.1  1.2  1.0  -1.3  1.8  -1.1  1.1  1.9  -1.1  2.7  1.4  -1.2  -1.1  1.2  1.2  -1.1  1.2  1.4  1.5  1.1  1.1  1.1  -1.1  -1.0  1.1  -1.1  1.3  1.4  1.8  1.1  -1.1  1.1  -1.1  -1.0  1.3  1.1  1.4  -1.0  1.1  -1.0  -1.0  1.3  -1.1  1.1  -1.2  -1.1  1.1  -1.1  1.1  -1.1  1.2  -1.1  -1.0  -1.2  -1.0  1.2  -1.0  1.2  -1.0  -1.1  -1.6  1.2  -1.2  1.0  1.1  1.1  1.4  -1.0  -1.0  1.1  1.3  -1.7  -1.2  -1.0  -1.2  1.1  -1.2  1.0  -1.0  -1.0  1.2  -1.6  1.1  1.1  -1.1  1.7  1.1  1.2  -1.2  1.1  1.2  -1.0  1.3  1.1  1.2  -1.2  1.3  1.1  -1.2  -1.0  -1.1  1.0  1.1  1.2  1.1  1.1  1.4  1.0  1.0  1.1  1.1  -1.0  1.1  1.2  -1.0  -1.1  1.1  1.0  -1.1  -1.0  1.2  1.0  1.3  -1.1  1.0  1.1  1.1  1.3  -1.0  -1.1  -1.0  -1.0  1.1  1.1  1.0  1.2  1.2  1.0  -1.0  1.1  1.4  -1.1  1.2  -1.2  -1.0  1.2  1.2  1.1  -1.2  1.1  1.0  1.0  1.1  -1.1  -1.0  1.1  1.4  1.1  1.3  1.1  -1.0  -1.1  -1.0  1.6  1.0  -1.0  -1.3  1.3  1.2  1.1  1.0  1.0  1.0  1.1  1.2  1.1  -1.0  1.1  1.1  -1.1  1.0  -1.1  -1.2  1.8  1.1  1.1  1.0  1.0  -1.0  1.2  1.2  -1.1  -1.1  -1.1  1.0  1.2  1.5  1.3  1.4  1.1  1.0  -1.1  -1.4  -1.0  -1.1  -1.2  -1.2  1.1  -1.1  1.2  1.1  1.1  1.1  1.1  1.1  -1.1  1.1  1.3  -1.6  -1.1  1.2  -1.1  -1.0  -1.4  1.1  -1.0  -1.1  1.2  -1.0  1.2  -1.1  -1.0  1.1  1.1  1.1  1.1  -1.0  1.1  1.0  -1.1  1.3  1.1  -1.1  -1.0  1.0  -1.0  -1.0  -1.1  1.1  1.1  -1.0  -1.0  1.1  -1.0  -1.1  -1.2  -1.1  -1.0  -1.2  1.4  -1.1  1.0  1.0  1.0  -1.0  -1.1  1.0  1.1  1.1  1.1  1.1  -1.1  -1.1  1.0  -1.0  -1.0  1.1  1.1  -1.0  1.1  1.2  -1.0  1.1  -1.1  1.1  1.1  1.1  1.3  1.0  -1.1  -1.1  1.4  -1.2  1.5  -1.1  1.0  1.1  1.0  1.1  -1.1  1.0  -1.0  -1.2  1.1  -1.1  1.1  1.0  -1.0  -1.0  -1.0  1.0  1.0  -1.2  -1.0  1.1  -1.0  1.0  1.1  -1.0  -1.1  -1.0  1.0  -1.0  -1.1  -1.1  1.2  -1.1  -1.1  -1.1  -1.1  1.1  1.3  1.1  1.1  1.0  1.1  1.1  1.1  1.2  -1.1  1.5  1.0  -1.2  1.1  1.2  1.1  1.2  1.1  -1.1  -1.1  1.1  1.0  -1.1  -1.1  1.1  -1.1  -1.0  -1.0  -1.1  -1.1  1.0  -1.4  -1.1  -1.1  -1.0  1.0  -1.0  1.0  1.1  -1.2  1.2  1.0  1.4  -1.2  -1.2  1.0  1.0  1.0  -1.1  -1.0  1.1  1.2  1.1  1.0  1.2  -1.1  1.1  1.1  -1.0  -1.1  -1.3  1.0  -1.1  -1.1  -1.1  -1.1  -1.0  -1.0  1.1  -1.0  -1.1  -1.1  -1.0  1.0  1.2  -1.1  -1.1  1.0  -1.1  -1.1  -1.0  -1.0  1.0  -1.0  -1.1  1.1  -1.1  1.2  -1.1  1.0  1.0  -1.1  1.1  1.1  1.0  -1.2  1.1  1.1  1.3  1.1  1.0  -1.1  1.1  -1.1  1.0  -1.1  -1.1  1.0  1.1  1.1  1.0  1.1  1.3  1.1  1.1  1.1  1.0  -1.0  -1.0  -1.1  1.0  -1.0  -1.1  1.1  1.1  1.1  1.1  -1.1  1.0  1.1  -1.0  -1.0  -1.2  1.0  1.1  -1.1  1.0  -1.0  1.0  1.1  -1.0  -1.0  1.0  -1.0  1.1  1.0  1.3  1.1  1.1  -1.0  1.1  -1.0  1.1  1.0  -1.0  -1.1  1.0  1.1  -1.0  1.1  -1.1  1.4  -1.0  1.0  1.1  -1.0  -1.1  -1.0  -1.0  -1.1  1.1  -1.0  -1.0  1.0  1.1  -1.1  1.0  -1.0  1.1  1.0  -1.0  -1.1  1.0  1.0  1.0  1.0  1.1  -1.0  -1.0  1.1  1.0  1.0  1.1  -1.0  -1.0  -1.1  -1.1  1.0  -1.1  -1.0  -1.1  1.1  1.0  1.0  1.0  -1.1  1.0  -1.0  -1.1  1.1  -1.0  1.0  1.0  -1.0  -1.0  -1.0  -1.0  1.0  1.1  -1.0  1.0  1.1  1.1  -1.0  1.0  1.0  1.0  1.0  1.1  1.1  1.0  1.1  1.1  -1.0  -1.0  1.1  1.1  -1.1  -1.1  1.0  -1.0  -1.0  -1.0  1.1  1.0  -1.1  -1.1  -1.1  -1.0  -1.0  -1.0  -1.1  -1.0  1.0  -1.1  1.0  -1.1  1.1  -1.1  1.0  1.0  1.0  1.0  1.0  1.0  1.0  -1.0  1.0  -1.0  1.0  1.0  1.0  -1.0  1.0  1.0  -1.0  1.0  1.0 | 0.4090  0.3894  0.7299  0.0060  0.7612  0.1049  0.0017  0.6027  0.0104  0.2493  0.3092  0.0007  0.1615  0.0305  0.6135  0.0006  0.0310  0.6624  0.0753  0.0017  0.0035  0.0007  0.2023  0.0515  0.0641  0.8595  0.0050  0.0107  0.0008  0.5155  0.6492  0.0977  0.0024  0.0011  0.6606  0.2396  0.5638  0.0240  0.0570  0.0538  0.0019  0.0185  0.0044  0.0063  0.4906  0.0019  0.0931  0.0092  0.3186  0.3511  0.5940  0.4485  0.1938  0.7111  0.8061  0.1558  0.4161  0.0110  0.3789  0.2036  0.1735  0.0144  0.0084  0.6063  0.7225  0.0797  0.0164  0.8588  0.2000  0.5035  0.9928  0.4274  0.1294  0.5565  0.2986  0.0226  0.0662  0.6782  0.2609  0.3480  0.5410  0.0241  0.0358  0.1934  0.3706  0.0065  0.0502  0.6861  0.3292  0.7250  0.0649  0.0570  0.5128  0.7807  0.2545  0.0175  0.0084  0.0081  0.0619  0.4188  0.6857  0.8088  0.1831  0.8181  0.1517  0.5790  0.0498  0.1307  0.3375  0.9678  0.1862  0.1201  0.0116  0.1923  0.2755  0.3097  0.0912  0.2697  0.0186  0.4004  0.9317  0.0530  0.2036  0.9584  0.0481  0.5007  0.0699  0.0259  0.4965  0.0123  0.4385  0.2367  0.6183  0.0430  0.0255  0.1971  0.4778  0.8855  0.9706  0.0351  0.0919  0.0822  0.0625  0.4168  0.4177  0.3628  0.6892  0.9970  0.2863  0.0255  0.3784  0.2274  0.5129  0.9309  0.4532  0.2641  0.1656  0.4668  0.6516  0.9588  0.0162  0.2204  0.0159  0.2719  0.0925  0.0406  0.3591  0.8027  0.3826  0.0242  0.7168  0.4621  0.0791  0.0624  0.4498  0.1190  0.9426  0.6714  0.1244  0.0533  0.1093  0.0817  0.1093  0.0528  0.0769  0.2738  0.0757  0.3995  0.0750  0.9376  0.1177  0.1033  0.8391  0.8151  0.1862  0.3740  0.2189  0.8306  0.9667  0.0619  0.5102  0.1928  0.5325  0.1065  0.5975  0.4411  0.4386  0.3243  0.3825  0.8814  0.1099  0.2120  0.7063  0.0527  0.4966  0.9014  0.4699  0.6722  0.1346  0.0976  0.4795  0.6124  0.0668  0.1303  0.1952  0.7525  0.5874  0.3828  0.6465  0.8052  0.5068  0.1177  0.7415  0.0378  0.5096  0.6312  0.4331  0.4405  0.6433  0.0997  0.1315  0.0292  0.1203  0.7390  0.0394  0.0554  0.1988  0.0430  0.1772  0.5584  0.8475  0.7260  0.3589  0.6369  0.5905  0.0645  0.6077  0.2497  0.0367  0.3713  0.9742  0.9745  0.2789  0.1006  0.2549  0.0779  0.9476  0.9315  0.2290  0.7646  0.9365  0.0761  0.3354  0.2771  0.2945  0.3669  0.3278  0.1719  0.0862  0.2249  0.2190  0.7530  0.4299  0.4534  0.6127  0.0401  0.0485  0.4912  0.7512  0.1458  0.6606  0.0415  0.3303  0.1408  0.7914  0.4520  0.0425  0.5326  0.0843  0.3081  0.1356  0.3387  0.2920  0.2719  0.4086  0.9797  0.3509  0.2471  0.1029  0.2978  0.1157  0.5919  0.3797  0.5182  0.2976  0.7407  0.2450  0.7271  0.1332  0.9110  0.6190  0.8807  0.7756  0.2947  0.0861  0.2504  0.4143  0.3294  0.3447  0.5430  0.2992  0.5939  0.1937  0.2414  0.7561  0.3560  0.8350  0.0731  0.9963  0.0735  0.8116  0.2894  0.1980  0.6050  0.1759  0.7449  0.3439  0.4831  0.0643  0.4158  0.7822  0.5105  0.3627  0.0642  0.1307  0.6344  0.1407  0.3126  0.0749  0.7746  0.9270  0.8891  0.0831  0.2959  0.3395  0.2819  0.2972  0.1215  0.6977  0.2570  0.0698  0.3949  0.1534  0.9381  0.3741  0.1649  0.1992  0.1769  0.1222  0.1322  0.0829  0.6899  0.5215  0.9954  0.5562  0.3024  0.4075  0.4339  0.1355  0.7160  0.9657  0.4416  0.2384  0.7225  0.0869  0.0976  0.8806  0.4147  0.2271  0.7247  0.1559  0.6326  0.0966  0.8140  0.0862  0.0784  0.7070  0.4902  0.3297  0.0999  0.7652  0.1090  0.8392  0.7393  0.2926  0.1202  0.6173  0.1352  0.3111  0.8627  0.7793  0.4007  0.0874  0.2436  0.1004  0.5290  0.9347  0.1136  0.2470  0.4615  0.0976  0.3066  0.9796  0.9535  0.4757  0.3828  0.5301  0.4286  0.4396  0.6111  0.2798  0.4349  0.9573  0.6560  0.8875  0.1698  0.7609  0.5604  0.1455  0.3700  0.1244  0.4285  0.7681  0.9761  0.7186  0.5222  0.1183  0.6132  0.8566  0.2195  0.3985  0.2337  0.8045  0.2658  0.2483  0.1535  0.2872  0.9070  0.9605  0.6658  0.9431  0.1217  0.1964  0.2971  0.1313  0.4326  0.9896  0.1519  0.1915  0.1924  0.2177  0.4795  0.8004  0.5160  0.2105  0.5573  0.6667  0.6652  0.1352  0.6292  0.2064  0.1244  0.4044  0.2206  0.2479  0.3516  0.7911  0.2620  0.2642  0.2604  0.1655  0.8059  0.4752  0.3354  0.8623  0.2770  0.3672  0.8370  0.3311  0.1759  0.7863  0.1843  0.2509  0.8740  0.1249  0.3245  0.4454  0.2653  0.7956  0.3199  0.9288  0.2359  0.2275  0.7297  0.2399  0.7771  0.9365  0.8084  0.7235  0.2575  0.4121  0.1976  0.7061  0.7770  0.2760  0.8890  0.6095  0.1511  0.1866  0.9767  0.2894  0.2491  0.5561  0.7091  0.8477  0.6470  0.6295  0.4376  0.8714  0.4711  0.5523  0.4057  0.4028  0.5571  0.2155  0.8034  0.9246  0.6706  0.4992  0.4225  0.7705  0.2986  0.5853  0.6454  0.4832  0.2295  0.4624  0.3009  0.6050  0.5126  0.5792  0.1994  0.4764  0.1673  0.1598  0.2513  0.3884  0.8665  0.2504  0.9417  0.3980  0.5431  0.6679  0.9778  0.2531  0.3992  0.5936  0.5335  0.5287  0.7667  0.9855  0.6511  0.6476  0.8400  0.3807  0.9669  0.2865  0.8187  0.7542  0.4378  0.8125  0.5885  0.7398  0.7886  0.8087  0.8103  0.7278  0.4053  0.5161  0.5501  0.3857  0.4188  0.2256  0.2224  0.4761  0.2788  0.9694  0.5170  0.2352  0.3155  0.1911  0.3152  0.2685  0.7383  0.4729  0.1938  0.2559  0.3931  0.2481  0.2150  0.5800  0.2831  0.5657  0.9614  0.2846  0.5184  0.4081  0.3201  0.7801  0.9897  0.2260  0.2132  0.7057  0.3361  0.6515  0.5008  0.9095  0.9833  0.8625  0.9841  0.7624  0.2214  0.3752  0.7388  0.3505  0.4952  0.2866  0.9129  0.7510  0.7111  0.4266  0.7692  0.7401  0.6017  0.2534  0.9748  0.2838  0.2805  0.4233  0.2231  0.9609  0.4495  0.6051  0.8076  0.4761  0.3515  0.5342  0.5644  0.4943  0.9497  0.3517  0.9835  0.3019  0.5464  0.8762  0.9580  0.3411  0.3123  0.4741  0.9690  0.2858  0.7733  0.7388  0.9305  0.8316  0.8252  0.4838  0.6776  0.3340  0.3794  0.3461  0.9319  0.8332  0.7606  0.2689  0.4879  0.5997  0.3228  0.5917  0.2584  0.6075  0.3052  0.9837  0.3161  0.5062  0.4142  0.9849  0.5025  0.5071  0.9415  0.5128  0.4959  0.8714  0.4898  0.3160  0.5959  0.4246  0.5728  0.7497  0.7925  0.8726  0.7637  0.7808  0.9949  0.3314  0.2934  0.5598  0.4976  0.3142  0.4765  0.9857  0.4292  0.9259  0.8230  0.3539  0.9888  0.3536  0.8490  0.7068  0.5574  0.7426  0.3658  0.6141  0.7870  0.4476  0.7398  0.3186  0.7604  0.4900  0.5198  0.7081  0.7524  0.5216  0.8848  0.4228  0.9723  0.7657  0.6512  0.6902  0.3743  0.6007  0.5209  0.6239  0.4248  0.8264  0.7141  0.5281  0.9101  0.4179  0.6568  0.9891  0.3713  0.5012  0.8375  0.7139  0.8881  0.6607  0.3992  0.4766  0.9149  0.3600  0.7467  0.9934  0.4035  0.6995  0.9098  0.8162  0.7421  0.5460  0.7795  0.8277  0.7338  0.9069  0.8726  0.5029  0.5655  0.9058  0.4018  0.4516  0.7500  0.4040  0.7841  0.4699  0.5998  0.7800  0.8338  0.8679  0.6198  0.9159  0.8889  0.4405  0.4775  0.7738  0.9592  0.9918  0.8586  0.9438  0.7666  0.9643  0.7154  0.4535  0.5669  0.5663  0.8017  0.6693  0.7604  0.7448  0.9027  0.7940  0.9020  0.5226  0.5765  0.9857  0.6112  0.4687  0.8330  0.8107  0.5973  0.6029  0.5317  0.5058  0.9122  0.9840  0.7896  0.6251  0.5034  0.8510  0.6160  0.7370  0.5624  0.8678  0.8252  0.7790  0.6496  0.9380  0.9561  0.6306  0.7462  0.6139  0.5865  0.6231  0.7995  0.6072  0.9083  0.8893  0.7991  0.9127  0.7636  0.7969  0.7695  0.7002  0.7549  0.8613  0.9271  0.9059  0.9380  0.9757  0.9553  0.9047  0.9919 |
