# Supplementary material for: An Exploration of Molecular Correlates Relevant to Radiation Combined Skin-Burn Trauma
Source: PLoS One. 2015 Aug 6;10(8):e0134827. doi: 10.1371/journal.pone.0134827 (PMC4527694; doi:10.1371/journal.pone.0134827)
Supplement: S2 Table — (DOC) [file pone.0134827.s003.doc]

**S2 Table**. List of 106 microRNA seed sequences identified using microarray to be differentially expressed in BURN mice compared to SHAM mice with respective fold change and p value of significance (p<0.05).

| **miRNA seed sequences** | **Microarray fold change** | **P value** | **miRNA seed sequences** | **Microarray fold change** | **P value** |
| --- | --- | --- | --- | --- | --- |
| miR-688  let-7a-1-star  miR-23b-star  miR-871-3p  miR-153  miR-350  miR-3065  miR-881  miR-196a-1-star  miR-495  miR-1306-3p  miR-1946a  miR-126-5p  miR-1264-5p  miR-199b  miR-669e-star  miR-196a-2-star  miR-3075  miR-138-1-star  miR-382-star  miR-742-star  miR-376c-star  miR-713  miR-107  miR-28c  miR-467h  miR-693-3p  miR-148a  miR-1962  miR-181a  miR-712-star  miR-34b-5p  miR-135a  miR-680  miR-192-star  miR-683  miR-3072-star  miR-20a-star  miR-133a  miR-219-5p  miR-3096b-3p  let-7c-2-star  miR-450a-1-star  miR-384-5p  miR-410  miR-208b  miR-1955-5p  miR-669p-star  miR-17-star  miR-504-star  miR-212-5p  miR-3087-star  miR-5128 | 1.8  1.6  1.6  1.4  1.6  2.8  7.0  1.3  1.7  9.4  1.9  1.3  1.4  -1.3  1.2  1.7  3.6  1.7  3.2  1.8  -1.3  1.5  1.3  1.4  -1.2  1.3  -1.2  -1.2  1.5  -1.2  -1.3  1.4  1.2  1.4  1.4  -1.3  1.4  1.3  -1.3  1.4  1.3  1.3  1.3  1.6  1.3  3.3  -1.3  1.7  1.3  1.3  1.2  1.4  -1.9 | 0.0000  0.0002  0.0006  0.0010  0.0011  0.0013  0.0018  0.0021  0.0022  0.0022  0.0033  0.0036  0.0038  0.0038  0.0039  0.0041  0.0048  0.0050  0.0057  0.0059  0.0064  0.0064  0.0067  0.0077  0.0079  0.0080  0.0081  0.0082  0.0083  0.0089  0.0093  0.0095  0.0097  0.0099  0.0102  0.0106  0.0110  0.0112  0.0114  0.0128  0.0155  0.0157  0.0158  0.0159  0.0166  0.0170  0.0181  0.0182  0.0191  0.0199  0.0203  0.0218  0.0222 | miR-302b  miR-92a  miR-1943  miR-5107  miR-362-5p  miR-219-3p  miR-7a-1-star  miR-377  miR-700  miR-1946b  miR-421-star  miR-103-1-star  miR-669h-3p  miR-214  miR-1897-5p  miR-34c  miR-376b-star  miR-30c-2-star  miR-195  miR-465c-5p  miR-21-star  miR-124  miR-466i-3p  miR-3068-star  miR-1897-3p  miR-496  miR-340-5p  miR-181a-2-star  miR-467e-star  miR-15a-star  miR-711  miR-543  miR-331-5p  miR-199b-star  miR-25-star  miR-122-star  miR-3105-3p  miR-1964-5p  miR-323-3p  miR-297a-star  miR-183  miR-744  miR-429  miR-125b-5p  miR-344e  miR-455  miR-3475  miR-466q  miR-3070b-5p  miR-467c-star  miR-1982.1  miR-204-star  miR-205 | - 1.2  -1.2  1.3  1.3  -1.2  1.2  3.2  1.6  1.9  1.2  -1.2  -1.3  2.4  1.3  1.2  1.3  1.4  1.5  1.4  1.7  1.2  -1.3  1.9  -1.1  -1.2  1.3  1.4  -1.2  1.9  1.3  1.5  -1.2  -1.3  -1.1  1.3  1.7  -1.2  1.5  1.2  2.0  1.2  1.2  -1.1  1.2  1.9  -2.6  1.2  2.3  -1.2  1.7  -1.2  1.4  -1.2 | 0.0223  0.0232  0.0242  0.0244  0.0247  0.0248  0.0257  0.0261  0.0261  0.0266  0.0267  0.0276  0.0285  0.0296  0.0297  0.0301  0.0303  0.0305  0.0307  0.0309  0.0314  0.0316  0.0324  0.0324  0.0347  0.0351  0.0360  0.0363  0.0384  0.0385  0.0390  0.0391  0.0392  0.0401  0.0425  0.0427  0.0432  0.0437  0.0439  0.0442  0.0444  0.0446  0.0451  0.0463  0.0468  0.0473  0.0479  0.0482  0.0484  0.0491  0.0491  0.0496  0.0497 |
