# Supplementary material for: An Exploration of Molecular Correlates Relevant to Radiation Combined Skin-Burn Trauma
Source: PLoS One. 2015 Aug 6;10(8):e0134827. doi: 10.1371/journal.pone.0134827 (PMC4527694; doi:10.1371/journal.pone.0134827)
Supplement: S3 Table — (DOC) [file pone.0134827.s004.doc]

**S3 Table**. List of 85 microRNA seed sequences identified using microarray to be differentially expressed in RI mice compared to SHAM mice with respective fold change and p value of significance (p<0.05).

| **miRNA seed sequences** | **Microarray fold change** | **P value** | **miRNA seed sequences** | **Microarray fold change** | **P value** |
| --- | --- | --- | --- | --- | --- |
| miR-410  miR-742-star  miR-196a-1-star  miR-31  miR-466n-3p  miR-138-1-star  miR-293-star  miR-467e-star  miR-214  miR-34c  miR-216b  miR-1264-5p  miR-1186  miR-767  miR-466h-3p  miR-3105-3p  miR-28c  miR-302d-star  miR-185  miR-142-5p  miR-688  miR-1191  miR-669p-star  miR-669e-star  miR-465c-5p  miR-467h  miR-344b  miR-144-star  miR-125b-5p  miR-101b  miR-103-2-star  miR-712-star  miR-3068-star  miR-331-5p  miR-3104-3p  miR-466i-5p  miR-1943  miR-26a-2-star  miR-574-5p  miR-130a  miR-669f-5p  miR-32-star  miR-499-star | 1.5  -1.3  1.7  -1.5  -1.6  3.5  1.2  2.5  1.4  1.4  -1.7  -1.3  -1.9  1.2  1.8  -1.3  -1.2  -1.3  -1.6  -1.5  1.3  -1.2  1.8  1.5  1.9  1.3  1.4  -1.6  1.2  1.2  1.3  -1.3  -1.1  -1.4  -1.2  2.3  1.3  -1.5  2.0  -1.9  1.4  1.2  1.3 | 0.0004  0.0017  0.0019  0.0026  0.0029  0.0034  0.0037  0.0044  0.0051  0.0052  0.0054  0.0057  0.0059  0.0064  0.0065  0.0070  0.0077  0.0089  0.0094  0.0096  0.0103  0.0105  0.0105  0.0122  0.0124  0.0126  0.0126  0.0129  0.0130  0.0135  0.0136  0.0139  0.0142  0.0152  0.0153  0.0153  0.0158  0.0158  0.0165  0.0171  0.0173  0.0174  0.0183 | miR-1912-star  miR-302b  miR-1912  miR-452-3p  miR-350  miR-503  miR-466q  miR-346  miR-1839-3p  miR-154  miR-344d-1-star  miR-21-star  miR-130b  miR-207  miR-3083-star  miR-3086-3p  miR-3082-5p  miR-1188-star  miR-99a-star  miR-669h-3p  miR-1950  miR-709  miR-350-star  miR-199b  miR-380-5p  miR-543  miR-34b-3p  miR-148a  miR-1893  miR-1982.1  miR-23b-star  miR-7b-star  miR-217-star  miR-1196  miR-135a  miR-574-3p  miR-669l  miR-3067-star  miR-187-star  miR-92a  miR-708-star  miR-141 | -1.2  1.2  -2.1  1.2  2.0  -1.3  2.6  1.3  2.9  1.3  -1.5  1.2  -1.7  1.3  1.3  -1.2  2.0  2.2  -1.2  2.3  1.2  -1.2  -2.3  1.2  1.2  -1.2  2.0  -1.2  1.3  -1.2  1.3  1.6  1.2  1.2  1.2  1.6  -1.2  1.3  1.8  -1.1  -1.2  1.2 | 0.0193  0.0200  0.0204  0.0211  0.0223  0.0224  0.0252  0.0253  0.0255  0.0268  0.0270  0.0293  0.0296  0.0330  0.0330  0.0330  0.0344  0.0372  0.0392  0.0394  0.0396  0.0398  0.0398  0.0411  0.0414  0.0416  0.0429  0.0439  0.0439  0.0443  0.0446  0.0453  0.0453  0.0456  0.0460  0.0460  0.0461  0.0468  0.0479  0.0486  0.0487  0.0498 |
