# Supplementary material for: An Exploration of Molecular Correlates Relevant to Radiation Combined Skin-Burn Trauma
Source: PLoS One. 2015 Aug 6;10(8):e0134827. doi: 10.1371/journal.pone.0134827 (PMC4527694; doi:10.1371/journal.pone.0134827)
Supplement: S4 Table — (DOC) [file pone.0134827.s005.doc]

**S4 Table**. List of 56 microRNA seed sequences identified using microarray to be differentially expressed in CI mice compared to SHAM mice with respective fold change and p value of significance (p<0.05).

| **miRNA seed sequences** | **Microarray fold change** | **P value** | **miRNA seed sequences** | **Microarray fold change** | **P value** |
| --- | --- | --- | --- | --- | --- |
| miR-669e-star  miR-467h  miR-466n-3p  miR-3090  miR-383-star  miR-452-3p  miR-742-star  miR-148a  miR-125b-5p  miR-466h-3p  miR-196a-1-star  miR-1186  miR-503  miR-331-3p  miR-138-1-star  miR-466q  miR-344d-1-star  miR-223  miR-1839-3p  miR-1264-5p  miR-153  miR-574-5p  miR-32-star  miR-125b-2-3p  miR-574-3p  miR-28c  miR-344e-star  miR-28b | 1.9  1.4  -1.7  1.4  1.6  1.3  -1.3  -1.3  1.3  1.9  1.6  -1.9  -1.3  -1.3  3.1  3.3  -1.7  1.6  3.6  -1.3  1.4  2.1  1.3  1.2  1.9  -1.2  -1.8  -1.3 | 0.0006  0.0007  0.0007  0.0008  0.0011  0.0017  0.0017  0.0019  0.0019  0.0024  0.0035  0.0044  0.0050  0.0060  0.0063  0.0065  0.0081  0.0084  0.0084  0.0092  0.0104  0.0107  0.0110  0.0116  0.0123  0.0144  0.0159  0.0162 | miR-346  miR-106a  miR-466i-5p  miR-302b  miR-34c  miR-350  miR-181a  miR-543  miR-154  miR-3572  miR-1912-star  miR-669o-5p  miR-410  miR-199b-star  miR-101b  miR-666-3p  miR-20a  miR-26b  miR-199a-3p  miR-3061-3p  miR-3100-3p  miR-375-star  miR-3109-star  let-7b-star  miR-1943  miR-1191  miR-690  miR-5128 | 1.3  -1.3  2.2  1.2  1.3  1.9  -1.2  -1.2  1.3  -1.4  -1.2  1.3  1.3  -1.1  1.2  -1.2  1.3  -1.2  1.2  -1.3  1.2  2.7  1.2  1.9  1.2  -1.2  1.8  -1.8 | 0.0164  0.0175  0.0185  0.0186  0.0226  0.0240  0.0241  0.0242  0.0255  0.0255  0.0259  0.0292  0.0305  0.0310  0.0351  0.0358  0.0367  0.0378  0.0394  0.0401  0.0406  0.0415  0.0425  0.0430  0.0430  0.0481  0.0485  0.0498 |
